# Alpha-synuclein overexpression induces epigenomic dysregulation of glutamate signaling and locomotor pathways

**DOI:** 10.1093/hmg/ddac104

**Published:** 2022-05-14

**Authors:** Samantha L Schaffner, Zinah Wassouf, Diana F Lazaro, Mary Xylaki, Nicole Gladish, David T S Lin, Julia MacIsaac, Katia Ramadori, Thomas Hentrich, Julia M Schulze-Hentrich, Tiago F Outeiro, Michael S Kobor

**Affiliations:** Department of Medical Genetics, Centre for Molecular Medicine and Therapeutics, British Columbia Children’s Hospital Research Institute, University of British Columbia, Vancouver, BC, V5Z 4H4, Canada; Department of Experimental Neurodegeneration, Center for Biostructural Imaging of Neurodegeneration, University Medical Center Göttingen, 37073 Göttingen, Germany; German Centre for Neurodegenerative Diseases (DZNE), 37075 Göttingen, Germany; Department of Experimental Neurodegeneration, Center for Biostructural Imaging of Neurodegeneration, University Medical Center Göttingen, 37073 Göttingen, Germany; Department of Experimental Neurodegeneration, Center for Biostructural Imaging of Neurodegeneration, University Medical Center Göttingen, 37073 Göttingen, Germany; Department of Medical Genetics, Centre for Molecular Medicine and Therapeutics, British Columbia Children’s Hospital Research Institute, University of British Columbia, Vancouver, BC, V5Z 4H4, Canada; Department of Medical Genetics, Centre for Molecular Medicine and Therapeutics, British Columbia Children’s Hospital Research Institute, University of British Columbia, Vancouver, BC, V5Z 4H4, Canada; Department of Medical Genetics, Centre for Molecular Medicine and Therapeutics, British Columbia Children’s Hospital Research Institute, University of British Columbia, Vancouver, BC, V5Z 4H4, Canada; Department of Medical Genetics, Centre for Molecular Medicine and Therapeutics, British Columbia Children’s Hospital Research Institute, University of British Columbia, Vancouver, BC, V5Z 4H4, Canada; Institute of Medical Genetics and Applied Genomics, University of Tübingen, 72076 Tübingen, Germany; Institute of Medical Genetics and Applied Genomics, University of Tübingen, 72076 Tübingen, Germany; Department of Experimental Neurodegeneration, Center for Biostructural Imaging of Neurodegeneration, University Medical Center Göttingen, 37073 Göttingen, Germany; German Centre for Neurodegenerative Diseases (DZNE), 37075 Göttingen, Germany; Max Planck Institute for Natural Sciences, 37075 Göttingen, Germany; Translational and Clinical Research Institute, Faculty of Medical Sciences, Newcastle University, Framlington Place, Newcastle-upon-Tyne, NE2 4HH, UK; Department of Medical Genetics, Centre for Molecular Medicine and Therapeutics, British Columbia Children’s Hospital Research Institute, University of British Columbia, Vancouver, BC, V5Z 4H4, Canada

## Abstract

Parkinson’s disease (PD) is a neurological disorder with complex interindividual etiology that is becoming increasingly prevalent worldwide. Elevated alpha-synuclein levels can increase risk of PD and may influence epigenetic regulation of PD pathways. Here, we report genome-wide DNA methylation and hydroxymethylation alterations associated with overexpression of two PD-linked alpha-synuclein variants (wild-type and A30P) in LUHMES cells differentiated to dopaminergic neurons. Alpha-synuclein altered DNA methylation at thousands of CpGs and DNA hydroxymethylation at hundreds of CpGs in both genotypes, primarily in locomotor behavior and glutamate signaling pathway genes. In some cases, epigenetic changes were associated with transcription. SMITE network analysis incorporating H3K4me1 ChIP-seq to score DNA methylation and hydroxymethylation changes across promoters, enhancers, and gene bodies confirmed epigenetic and transcriptional deregulation of glutamate signaling modules in both genotypes. Our results identify distinct and shared impacts of alpha-synuclein variants on the epigenome, and associate alpha-synuclein with the epigenetic etiology of PD.

## Introduction

Alpha-synuclein (aSyn) plays crucial roles in neurodevelopment, neuronal health, and synaptic transmission (1). Mutations, multiplications, and single nucleotide polymorphisms (SNPs) in *SNCA*, the gene encoding aSyn, are associated with Parkinson’s disease (PD), an age-associated, and therefore increasingly prevalent, neurological disorder (2,3). *SNCA* variants have also been linked to several other neurodegenerative conditions, including Lewy body dementia (LBD), multiple system atrophy (MSA), and Alzheimer’s disease (4,5). The full range of functions of aSyn are still unclear, as a number of different cellular roles have been reported, including SNARE complex assembly, regulation of neuronal differentiation, glucose level, and dopamine biosynthesis, as well as modulation of calmodulin activity/G protein-coupled receptor kinase activity (1). aSyn point mutations can impair one or several of these processes, and thus disrupt neuronal health. Interestingly, we recently reported that nuclear localization and phosphorylation modulate the pathological effects of aSyn (6). However, the molecular mechanisms leading to PD may differ according to the variant. For example, the A30P mutation occurs in the membrane binding domain of aSyn, which may affect its ability to act as a presynaptic chaperone and interact with membrane-bound receptors (7). Although A30P aSyn appears less likely to aggregate than wild-type (WT) aSyn, this mutation can still result in PD, possibly due to a loss of aSyn function (7). Conversely, multiplications of the *SNCA* gene can lead to excess protein production, promoting aggregations and fibrils that impair synaptic function and can lead to neuronal death (8). Duplications and triplications of *SNCA* have been reported in patients with familial PD, LBD, and MSA (2,4).

Despite progress in the understanding the relations between aSyn dysfunction and PD, much remains to be elucidated. For example, although aSyn- and PD-associated transcriptional deregulation have been demonstrated in cell culture, animal models, and human patients, the mechanisms underlying these transcriptional changes are not yet fully understood (9–16). As different aSyn mutations can lead to PD through different mechanisms, it is also important to understand which aspects of these variants are unique and which are shared. Studies by our group and others have shown that expression of WT or A30P mutant aSyn in human neurons is associated with DNA damage and transcriptional dysregulation of genes involved in cell survival and DNA repair, which may be mediated by altered histone H3 acetylation (9,10). In addition, altered DNA methylation (DNAm) patterns have been observed in both the *SNCA* gene itself and genome-wide in blood, saliva, and brain of PD patients (17–21). Taken together, these observations suggest that genome-wide transcriptional and epigenetic dysregulation may be consequences of aSyn overexpression and could potentially be involved in PD susceptibility and pathogenesis.

Epigenetic studies of PD provide the opportunity to assess genetic and environmental influences on disease risk concurrently (22–28). DNA methylation (DNAm), which refers to the attachment of a methyl group to DNA on the 5′ carbon of cytosine, frequently in the context of cytosine-guanine (CpG) dinucleotides, is a well-studied epigenetic mark that changes during development and is influenced by genes and environment (22–25,29,30). Although DNAm patterns can be unrelated to mRNA expression patterns or laid down as a consequence of gene expression, in some cases DNAm can also impact transcription by altering the ability of transcription factors to bind to gene regulatory regions, changing chromosomal interactions, and influencing splicing (22,29–31). This can alter the regulation of pathways involved in disease susceptibility (32–34). In addition, DNAm patterns can be useful as biomarkers of aging, disease states, or exposures regardless of association with transcription (35–41).

Although DNAm is influenced by a myriad of factors that differ between individuals, the existing literature on DNAm in PD has primarily adopted a case–control approach (17–21). In addition, the role of DNA hydroxymethylation (DNAhm), which accounts for up to 40% of all modified cytosines in brain tissue, remains unclear (42). DNAhm is an oxidized form of DNAm, and an intermediate of the DNA demethylation process; however, DNAhm has also been suggested to be a stable, independent epigenetic mark in the brain (43). The stability of DNAhm and its ability to influence transcription by eliminating DNAm–protein interactions, introducing DNAhm–protein interactions, altering chromatin state, and altering splicing suggest that it could impact the etiology of neurological diseases (44,45). It is important to distinguish DNAhm from DNAm, as conventional bisulfite conversion techniques measure both DNAhm and DNAm in one compound signal, which may bias interpretations (46). DNAhm is stable in postmortem formalin-fixed tissue, and some initial studies characterized DNAhm patterns in the brains of deceased PD patients (47). Bulk DNAhm levels are unchanged in the cortex, substantia nigra, and brainstem of PD patients, while DNAhm levels and expression of TET2, the enzyme that forms this mark, are elevated in neurons of the prefrontal cortex of PD patients (48,49). Importantly, DNAm and DNAhm patterns can be influenced by genetics, and genetic alterations at loci, such as *SNCA*, influence PD susceptibility, suggesting that it will be important to incorporate genetic considerations into future epigenetic studies of PD (50–52). To date, only a few studies have assessed the impact of genetic background on DNAm patterns in PD (53–55).

Accounting for the range of genetic, environmental, and lifestyle factors that can influence DNAm and DNAhm are major challenges in human epigenetic studies of PD (22,23,29,50). Furthermore, the use of postmortem tissue to study PD also typically limits the analysis to cases of advanced disease. Model systems, such as rodents or cell culture, can help to address these issues, providing contexts in which both genetics and environment can be tightly controlled, and allowing the examination of early impacts of neurological disease in the brain.

In this study, we characterized the influence of two molecularly distinct aSyn variants, WT and A30P aSyn, on the DNA methylome and hydroxymethylome of human dopaminergic neurons. We profiled genome-wide DNAm and DNAhm patterns in three groups of Lund human mesencephalic (LUHMES) cells differentiated into dopaminergic neurons: control LUHMES cells, LUHMES cells overexpressing WT aSyn at levels similar to those seen in *SNCA* multiplication carriers (WT aSyn cells), and LUHMES cells overexpressing A30P aSyn (A30P aSyn cells). In addition, we integrated our DNAm and DNAhm data with transcriptomic data from the same cells, incorporating H3K4me1 ChIP-seq to score DNA(h)m changes across gene regulatory features (9). WT and A30P aSyn expression were associated with thousands of DNA(h)m changes, particularly affecting genes that regulate locomotor behavior and glutamate signaling pathways. These observations suggested that familial PD-associated aSyn mutations have widespread epigenomic effects, and may contribute to molecular and transcriptional dysregulation associated with the etiology and heterogeneity of PD.

## Results

### aSyn overexpression altered genome-wide DNAm patterns in dopaminergic neurons

**Figure 1:**
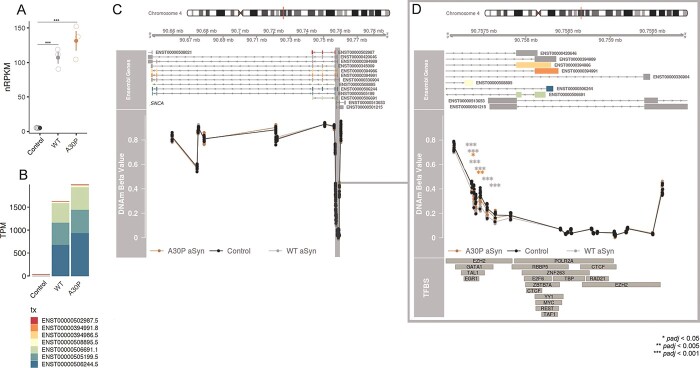
aSyn overexpression was associated with decreased DNAm at the *SNCA* 5′ UTR. (A) Gene-level *SNCA* mRNA expression in nRPKM. *p*-values: *DESeq2* Wald test with Benjamini–Hochberg adjustment. WT aSyn vs. control and A30P aSyn vs. control *padj* < 2.2 × 10^−308^. (B) Transcript-level *SNCA* mRNA expression in TPM. See Supplementary Material, Table S1 for statistics. (C) Top: hg19 coordinates are shown for the *SNCA* gene, with Ensembl transcripts below. Boxes represent exons, lines represent introns. Bottom: DNAm beta values are shown for each sample, colored by genotype. Only EPIC probes specific to endogenous *SNCA* are shown. Control cells: *n* = 7 biological replicates; WT aSyn cells: *n* = 8; A30P aSyn cells: *n* = 8. (D) hg19 coordinates, DNAm beta values, and transcription factor binding sites for the *SNCA* 5′ UTR/intron 1/TSS upstream region. *p*-values: *limma* empirical Bayes moderated *t* test with Benjamini–Hochberg adjustment. Bottom: ENCODE transcription factor ChIP-seq clusters.

We used differentiated LUHMES cells as a model system in which to evaluate the epigenome-wide impacts of aSyn expression in dopaminergic neurons. Genome-wide DNAm and DNAhm were analyzed in the same cells as used in a previously published transcriptomic analysis by our group (GSE89115, GSE181126) (9). Previously, we verified dopaminergic neuronal differentiation in these cells by immunostaining, which demonstrated the presence of tyrosine hydroxylase (*TH*)-positive axonal and dendritic networks, as well as endogenous aSyn expression (Supplementary Material, Fig. S1). Proliferating LUHMES cells were infected with lentiviral constructs containing IRES-GFP, WT aSyn-IRES-GFP, or A30P aSyn-IRES-GFP, and positive clones were selected by fluorescence activated cell sorting (FACS). As expected, the levels of *SNCA* mRNA and aSyn protein expression were higher in WT aSyn cells and A30P aSyn cells than in control cells, as determined by RNA-seq (approximately 4-fold in both genotypes) (Fig. 1A, B, Supplementary Material, Table S1) and immunoblotting (approximately 6-fold in both genotypes, see ref. 9). Elevated *SNCA* mRNA expression was primarily driven by three transcripts in WT and A30P aSyn cells (Fig. 1B, Supplementary Material, Table S1).

To investigate the impact of WT and A30P aSyn expression on genome-wide DNAm patterns, we assessed DNAm differences between control, WT aSyn, and A30P aSyn cells at 813589 EPIC probes. An effect size threshold of |delta beta| ≥ 0.05 was selected to be well above technical noise (maximum 2.2% RMSE between technical replicates), and probes were considered to be statistically significant at a Benjamini–Hochberg adjusted *p* (*padj*) ≤ 0.05, corrected for multiple comparisons. First, we examined whether overexpression of aSyn was associated with reduced DNAm at the first intron of *SNCA*, as reported previously in PD patients (20,21). We expected DNAm to be lower at this region in aSyn-expressing LUHMES cells as intron 1 has been established as a putative promoter for *SNCA* containing GATA binding sites, and enzymatic removal of DNAm in this region is associated with increased levels of *SNCA* mRNA and protein expression (56,57). In addition, to specifically examine DNAm levels at the endogenous gene without confounding by CpGs also located on the transgene, we removed five CpGs mapping to the cDNA sequence used in the aSyn constructs (NM_000345) and the remaining 35 *SNCA* CpGs are shown in Fig. 1C, D. As expected, both aSyn genotypes had significantly lower levels of DNAm at several CpGs in the region corresponding to intron 1 for the majority of *SNCA* transcripts, and upstream of the transcription start site (TSS) for several other transcripts (Fig. 1C, D). This region covered binding sites for GATA1, EZH2, TAL1, and EGR1 (Fig. 1D).

**Figure 2:**
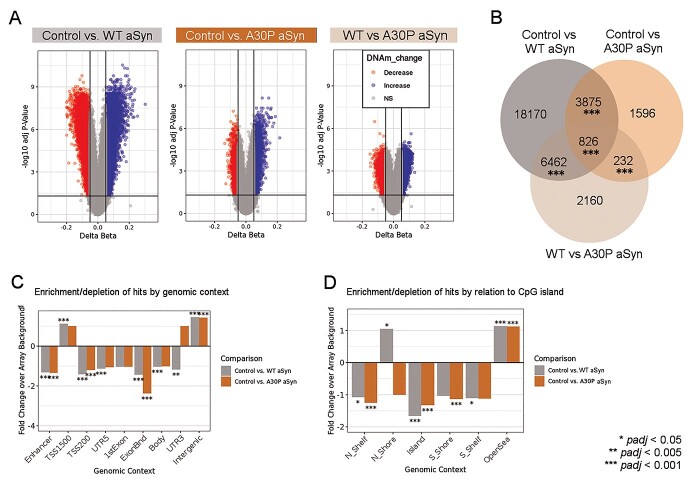
Overexpression of WT and A30P mutant aSyn altered the DNA methylome of dopaminergic neurons. (A) Volcano plots comparing DNAm patterns between control (*n* = 7 biological replicates) and WT aSyn (*n* = 8) LUHMES cells, control and A30P aSyn (*n* = 8) LUHMES cells, and WT aSyn and A30P aSyn LUHMES cells. Colored points passed thresholds of delta beta ≥0.05 and *padj* ≤ 0.05 (*limma* empirical Bayes moderated *t* test with Benjamini–Hochberg adjustment). (B) Number of differentially methylated probes unique to each comparison and probes shared between comparisons (*padj* < 0.001, 10 000 permutations). (C) Relative enrichment/depletion of differentially methylated sites across genomic contexts, permuted against array background (10 000 iterations). (D) Relative enrichment/depletion of differentially methylated sites by relation to CpG island, permuted against array background (10 000 iterations).

We next assessed genome-wide DNAm changes induced by overexpression of each aSyn variant. First, we used a site-specific approach, with linear modeling applied to all CpGs passing quality control (QC) filters (Fig. 2, Table 1). In comparison to controls, WT aSyn cells had 18 521 sites with decreased DNAm and 10 812 sites with increased DNAm (*padj* ≤ 0.05) (Fig. 2A, Table 1, Supplementary Material, Table S2). In A30P aSyn cells, 3091 probes had decreased DNAm and 3438 probes had increased DNAm compared to control cells (Fig. 2A, Table 1, Supplementary Material, Table S3). In comparison to WT aSyn cells, A30P aSyn cells had 3014 sites with decreased DNAm and 6666 sites with increased DNAm (Fig. 2A, Supplementary Material, Table S4). DNAm changes in all comparisons were significantly skewed in one direction, and the bias in effect direction was seen across all genomic features (*p* < 2.2e^−16^) (Supplementary Material, Table S5). To confirm an example from our findings by pyrosequencing, we selected a region of the *TUBA8* gene that included the top two probes with the greatest changes in DNAm across both genotypes; differential DNAm at these two positions, one additional position on the array, and four positions not on the array were all confirmed (Table 1, Supplementary Material, Fig. S2, Supplementary Material, Table S5).

**Table 1:** Top 5 unique and shared differentially methylated probes for each comparison, ranked by delta beta

**Control vs. WT aSyn unique**					
**Chr**	chr5	chr4	chr7	chr4	chr1
**Pos**	42 994 123	151 462 241	44 614 305	151 503 546	57 884 286
**Probe ID**	cg14979301	cg09064570	cg05758978	cg22265605	cg01074356
**WT aSyn adjusted *p*-value**	2.54e^−06^	5.36e^−07^	0.000236254	4.86e^−07^	0.000191117
**WT aSyn delta beta**	−0.1995039	0.19595166	−0.186602	0.1851907	0.18111904
**A30P aSyn adjusted *p*-value**	0.09078044	0.12926655	0.05169001	0.25104086	0.12524333
**A30P aSyn delta beta**	−0.0646483	0.0490894	−0.1084993	0.03763804	0.06857821
**Gene**		*LRBA*	*DDX56*	*LRBA*	*DAB1*
**Genomic context**		Body	TSS200	Body	5′ UTR
**Relation to CpG island**	N_Shore	OpenSea	S_Shore	N_Shore	N_Shelf
**Control vs. A30P aSyn unique**					
**Chr**	**chr10**	**chr22**	**chr21**	**chr14**	**chr1**
**Pos**	131 694 689	45 809 619	44 915 811	65 006 281	245 755 356
**Probe ID**	cg26275858	cg05072848	cg14177086	cg21035222	cg18199753
**WT aSyn adjusted *p*-value**	0.914887877	0.006470176	0.265419174	0.008640058	0.065585536
**WT aSyn delta beta**	0.00315511	0.04852953	−0.0369103	0.04451941	−0.0481363
**A30P aSyn adjusted *p*-value**	7.93e^−05^	1.91e^−05^	0.002633198	2.86e^−07^	0.000666635
**A30P aSyn delta beta**	−0.1182818	0.11255114	−0.1123709	0.10928006	−0.1059641
**Gene**	*EBF3*	*SMC1B*		*HSPA2*	*KIF26B*
**Genomic context**	Body	TSS200		TSS1500	Body
**Relation to CpG island**	Island	Island	OpenSea	N_Shore	S_Shore
**Control vs. WT aSyn and Control vs. A30P aSyn shared**					
**Chr**	**chr22**	**chr22**	**chr10**	**chr12**	**chr22**
**Pos**	18 593 586	18 593 441	94 455 710	12 849 159	18 593 609
**Probe ID**	cg27391512	cg25835669	cg07856667	cg13417420	cg08715837
**WT aSyn adjusted *p*-value**	1.69e^−08^	1.38e^−09^	2.08e^−09^	1.17e^−07^	2.38e^−08^
**WT aSyn delta beta**	0.30304448	0.23725831	0.23219774	0.25294376	0.2215025
**A30P aSyn adjusted *p*-value**	4.62e^−06^	1.44e^−07^	7.46e^−07^	0.0005165	1.06e^−05^
**A30P aSyn delta beta**	0.21622949	0.18841661	0.16977899	0.13573927	0.1532297
**Average delta beta**	0.25963699	0.21283746	0.20098836	0.19434151	0.1873661
**Gene**	*TUBA8*	*TUBA8*		*GPR19*	*TUBA8*
**Genomic context**	5′ UTR	TSS200		TSS200	5′ UTR
**Relation to CpG island**	Island	Island	Island	Island	Island

The proportion of differentially methylated probes shared across all comparisons and any two of three comparisons was greater than expected by chance, and in most cases, the differences from controls were more pronounced for WT aSyn cells than for A30P aSyn cells (*padj* < 0.001, 10 000 permutations) (Fig. 2B, Table 1). Interestingly, WT and A30P aSyn overexpression primarily affected CpGs outside of genes and enhancers as defined by H3K4me1 ChIP-seq (Fig. 2C). However, differentially methylated probes were enriched in TSS1500 regions for both genotypes (statistically significant in WT only), and trends for differentially methylated probe feature enrichment differed between genotypes for 3′ UTRs and CpG North Shores (Fig. 2C, D).

Following the investigation of differential DNAm patterns at individual CpG sites, we additionally examined genotype-dependent DNAm differences at co-methylated regions (CMRs) (Supplementary Material, Fig. S3). CMRs are defined as regions with correlated DNAm across individuals, and such regional patterns of DNAm are more likely to be biologically meaningful than DNAm at single CpGs (58). We defined 57 941 custom DNAm CMRs in LUHMES neurons and applied linear modeling to the composite beta values calculated for each CMR, representing a weighted average of CMR probe DNAm levels. Similar trends were seen in CMR analysis and site-specific analysis; the number of DNAm changes was greater in WT aSyn cells vs. control (3755 CMRs with *padj* ≤ 0.05 and |delta composite beta| ≥ 0.05) than in either A30P aSyn cells vs. control (548 CMRs) or A30P aSyn cells vs. WT aSyn cells (765 CMRs) (Supplementary Material, Fig. S3A, B). Approximately 40% of the total differentially methylated probes discovered with the site-specific approach mapped to CMRs in any comparison, and overlaps between differentially methylated CMRs and individual CpGs were more likely than expected by chance (*padj* < 0.001, 10 000 permutations) (Supplementary Material, Fig. S3A). Trends for differential CMR methylation across gene features were also similar to the site-specific approach (Supplementary Material, Fig. S3C, D). A CMR covering the *SNCA* first intron region was included among the differentially methylated CMRs in the control vs. WT aSyn and control vs. A30P aSyn comparisons, passing the composite delta beta cutoff only in WT aSyn neurons (chr4: 90757139–90 757 814; control vs. WT aSyn *padj* = 9.96e^−10^, delta composite beta = −0.083; control vs. A30P aSyn *padj* = 0.0029, delta composite beta = −0.032).

### aSyn overexpression altered genome-wide DNAhm patterns to a lesser extent than DNAm patterns

**Figure 3:**
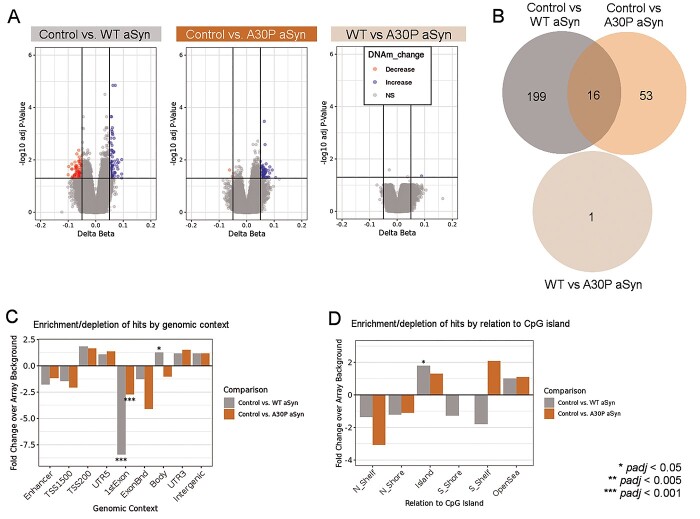
WT and A30P aSyn overexpression were correlated with increased DNAhm levels in dopaminergic neurons. (A) Volcano plots comparing DNAhm patterns between control (*n* = 7 biological replicates) and WT aSyn (*n* = 8) LUHMES cells, control and A30P aSyn (*n* = 8) LUHMES cells, and WT aSyn and A30P aSyn LUHMES cells. Colored points passed thresholds of delta beta ≥0.05 and *padj* ≤ 0.05 (*limma* empirical Bayes moderated *t* test with Benjamini–Hochberg adjustment). (B) Number of differentially hydroxymethylated probes unique to each comparison and probes shared between comparisons. All *padj* = 1 (10 000 permutations). (C) Relative enrichment/depletion of differentially hydroxymethylated sites across genomic contexts, permuted against array background (10 000 iterations). (D) Relative enrichment/depletion of differentially hydroxymethylated sites by relation to CpG island, permuted against array background (10 000 iterations).

After assessing genome-wide DNAm alterations, we examined differential DNAhm at 233440 sites that passed our detection threshold of 3.6% (calculated according to the 95% quantile of negative hydroxymethylation (hmC) values after subtraction; see Methods). Fewer changes were observed in DNAhm for each comparison compared to DNAm, most of which were increases; these increases in DNAhm were significant when tested for skewness (*p* < 2.2e^−16^) (Fig. 3, Table 2, Supplementary Material, Tables S6,S7). Only one probe was differentially hydroxymethylated in comparison between WT aSyn and A30P aSyn cells (cg11833293 in the *SHANK2* 3′ UTR, *padj* = 0.045, delta beta = 0.089) (Fig. 3A, B). The overlaps of 16 CpG sites between control vs. WT aSyn and control vs. A30P aSyn were not greater than expected by chance (Fig. 3B, *padj* > 0.05, 10 000 permutations). Both genotypes also had fewer differentially hydroxymethylated probes in the first exon than expected by chance (Fig. 3C).

**Table 2:** Top 5 unique and shared differentially hydroxymethylated probes for each comparison, ranked by delta beta

**Control vs. WT aSyn unique**					
**Chr**	chr11	chr1	chr11	chr5	chr15
**Pos**	11 959 869	157 010 623	119 191 624	134 825 791	99 329 769
**Probe ID**	cg21029705	cg11184358	cg12762029	cg01889143	cg08307952
**WT aSyn adjusted *p*-value**	3.71e^−02^	1.80e^−02^	0.009721248	4.94e^−02^	0.034644699
**WT aSyn delta beta**	−0.100144845	−0.098018504	0.0944902	0.094449407	0.09426821
**A30P aSyn adjusted *p*-value**	0.8044695	0.2341719	0.2673974	0.2917405	0.6017781
**A30P aSyn delta beta**	−0.0315162	−0.06167421	0.03536456	0.0533201	0.02102634
**Gene**	*USP47*	*ARHGEF11*	*MCAM*		*IGF1R*
**Genomic context**	Body	Body	3′ UTR		5′ UTR
**Relation to CpG island**	OpenSea	OpenSea	Island	Island	OpenSea
**Control vs. A30P aSyn unique**					
**Chr**	**chr3**	**chr19**	**chr6**	**chr21**	**chr2**
**Pos**	173 001 443	54 720 832	20 697 104	33 267 395	192 759 994
**Probe ID**	cg03417233	cg12555719	cg25475839	cg04815049	cg14971567
**WT aSyn adjusted *p*-value**	0.538342	0.2605252	0.1508893	0.16631	0.4370172
**WT aSyn delta beta**	0.0383568	0.03308958	0.0366181	0.0432397	0.01959213
**A30P aSyn adjusted *p*-value**	4.75e^−02^	2.44e^−02^	0.018507248	4.64e^−02^	0.024865866
**A30P aSyn delta beta**	0.105273858	0.092858583	0.084182475	0.080982712	0.078224072
**Gene**	*RP11-324C10.1*	*LILRB3*	*CDKAL1*	*HUNK*	*AC098617.2*
**Genomic context**	5′ UTR	3′ UTR	Body	Body	5′ UTR
**Relation to CpG island**	OpenSea	OpenSea	OpenSea	OpenSea	OpenSea
**Control vs. WT aSyn and Control vs. A30P aSyn shared**					
**Chr**	**chr12**	**chr16**	**chr7**	**chr8**	**chr7**
**Pos**	56 121 485	24 834 981	1 281 200	108 510 324	158 863 255
**Probe ID**	cg22249612	cg14908168	cg02471897	cg16318522	cg00128168
**WT aSyn adjusted *p*-value**	2.23e^−02^	9.93e^−03^	1.76e^−02^	3.76e^−02^	3.98e^−02^
**WT aSyn delta beta**	0.080308633	0.078234846	0.059501066	0.053512554	0.054876613
**A30P aSyn adjusted *p*-value**	3.19e^−02^	3.88e^−02^	2.32e^−02^	0.02916036	3.19e^−02^
**A30P aSyn delta beta**	0.083408157	0.059676009	0.064654602	0.067996928	0.066623524
**Average delta beta**	0.08185839	0.06895543	0.06207783	0.06075474	0.06075007
**Gene**	*CD63*	*TNRC6A*		*ANGPT1*	*VIPR2*
**Genomic context**	Body	Body		TSS200	Body
**Relation to CpG island**	N_Shore	OpenSea	Island	OpenSea	OpenSea

When defining CMRs in the DNAhm data and applying linear modeling, we also found greater increases in CMR DNAhm in control vs. WT aSyn and control vs. A30P aSyn comparisons (Supplementary Material, Fig. S4). Although fewer CMRs were found in DNAhm data overall, differentially hydroxymethylated CMRs were also more likely to overlap with differentially hydroxymethylated CpGs from site-specific analysis than expected by chance (Supplementary Material, Fig. S4A).

### WT and A30P aSyn altered DNAm at locomotor and glutamate signaling pathway genes

**Figure 4:**
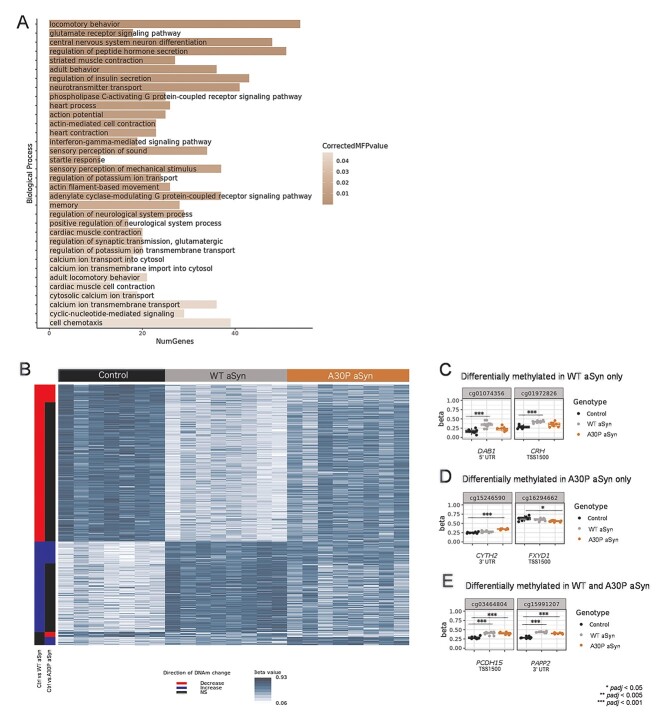
Differentially methylated genes in both genotypes were enriched for locomotor behavior and glutamate signaling functions. (A) GO biological process terms enriched at multifunctionality-corrected, multiple test-corrected hypergeometric *p*-value (CorrectedMFPvalue) < 0.05 in genes shared between control (*n* = 7 biological replicates) vs. WT aSyn (*n* = 8) and control vs. A30P aSyn (*n* = 8) DNAm analyses. (B) Heat map showing beta values for all probes annotated to the ‘locomotory behavior’ pathway and differentially methylated in at least one comparison. Row labels (left to right): probes that passed significance thresholds (absolute delta beta ≥0.05 and *padj* ≤ 0.05, *limma* empirical Bayes moderated *t* test with Benjamini–Hochberg adjustment) in control vs. WT aSyn comparison, probes that passed significance thresholds in control vs. A30P aSyn comparison. (C) Representative examples of 353 total locomotory behavior-related CpG sites differentially methylated only in WT aSyn cells (*DAB1 padj* = 1.91e^−4^, *CRH padj* = 9.34e^−5^). (D) Representative examples of 22 total locomotory behavior-related CpG sites differentially methylated only in A30P aSyn cells (*CYTH2 padj* = 1e^−5^, *FXYD1 padj* = 0.028). (E) Representative examples of 67 total locomotory behavior-related CpG sites differentially methylated in both genotypes (*PCDH15* WT aSyn *padj* = 7.8e^−6^, *PCDH15* A30P aSyn *padj* = 8.61e^−5^, *PAPP2* WT aSyn *padj* = 1.68e^−9^, *PAPP2* A30P aSyn *padj* = 3.55e^−7^). *p*-values: *limma* empirical Bayes moderated *t* test with Benjamini–Hochberg adjustment.

To identify possible functional consequences associated with the DNAm and DNAhm changes in each genotype, we performed Gene Ontology (GO) enrichment analysis on all differentially methylated or hydroxymethylated genes within each comparison using over-representation analysis in ermineR (59). Thirty-four GO biological processes were enriched in differentially methylated sites shared between the control vs. WT aSyn and control vs. A30P aSyn comparisons (Fig. 4A, *padj* < 0.05). A total of 442 CpG sites were annotated to the top GO term, ‘locomotory behavior,’ and differentially methylated in at least one genotype (420 sites in WT aSyn cells and 89 sites in A30P aSyn cells) (Fig. 4B–E). A total of 129 differentially methylated sites were annotated to the second highest ranking GO term, ‘glutamate receptor signaling pathway’ (127 sites in WT aSyn cells and 18 sites in A30P aSyn cells) (Supplementary Material, Fig. S5). No significant multifunctionality-corrected enrichments were observed for differentially methylated or hydroxymethylated sites in any of the other groups.

### A subset of WT aSyn-induced DNAm and DNAhm changes were also observed in WT aSyn transgenic mice

To assess the relevance of our findings *in vivo*, the differential DNA(h)m results from control vs. WT aSyn LUHMES cells were compared to those in the hippocampus of 12-month-old mice with high-level (sixfold over normal) expression of WT human aSyn from a BAC construct (14). Hippocampal DNAm and DNAhm levels were profiled by reduced representation bisulfite sequencing (RRBS) with the same paired bisulfite and oxidative bisulfite conversion approach as used for LUHMES cells (Schaffner et al., manuscript in preparation). At the DNAm level, 4037 of 8391 differentially methylated genes in WT aSyn LUHMES cells were also covered in the mouse RRBS data. Of these, 44 genes were differentially methylated in control vs. WT aSyn mice (*padj* ≤ 0.05 and |delta beta| ≥ 0.1, chosen to match the minimum 10× read coverage filter applied during RRBS QC). The number of genes differentially methylated within this subset of 4037 in mice was greater than expected by chance (*padj* < 0.001, 10 000 permutations). These included *Grm3*, encoding a metabotropic glutamate receptor, as well as several genes encoding transcriptional regulators, cell adhesion proteins, glucose and ion transporters, and cell signaling proteins (Supplementary Material, Table S8). At the DNAhm level, 50 of 136 differentially methylated LUHMES cell genes were covered by RRBS, with 9 being differentially hydroxymethylated (greater than expected by chance, *padj* < 0.001, 10 000 permutations). Differentially hydroxymethylated genes in WT aSyn mice and LUHMES cells had similar functions to differentially methylated genes, including transcription regulation, cell adhesion and signaling, as well as a folate transporter (*Slc19a1*) and vacuolar ATPase (*Atp6v0a1*; Supplementary Material, Table S9).

### aSyn impacted epigenetic and transcriptional regulation of glutamate, NOTCH, insulin, PDGF, and SHH signaling network genes

We next queried genes and pathways associated with changes in both the epigenome and the transcriptome in WT and A30P aSyn LUHMES neurons. These loci represent candidates where DNAm and/or DNAhm may be involved in regulation of gene expression, and their identification is important to understand the molecular etiology of PD. First, we identified CpG sites with alterations in both DNAm and DNAhm; in both genotypes, there were more CpGs with changes to both modifications than expected by chance (109 CpGs in WT aSyn cells, 34 CpGs in A30P aSyn cells, *padj* < 0.001, 1000 permutations) (Supplementary Material, Fig. S6). This was likely to be a biological effect, as DNAhm can be an intermediate of DNA demethylation; thus, CpG sites with increased DNAhm are likely to have decreased DNAm (60). Indeed, all of the CpGs with changes to both DNAm and DNAhm had opposite effect directions for each modification (Supplementary Material, Fig. S6).

Next, we compared the DNAm and DNAhm hits identified in this analysis against our previously published differential expression results from the same cells (863 differentially expressed genes in WT aSyn cells and 1315 genes in A30P aSyn cells, |log2FC| > 0.5 and *padj* < 0.01; validated by qPCR, see ref. 9). Of 10 798 genes with data for all three modifications in WT aSyn cells, seven showed differential DNAm, DNAhm, and expression, including an ionotropic glutamate receptor (*GRIK2*) (Supplementary Material, Table S10). In A30P cells, two of 10 831 genes had concurrent changes to these modifications: *TSPEAR* (cg04837104 and cg11631644 with altered DNAm, delta beta = 0.07 and 0.06, *padj* = 0.006 and 0.003; cg12061357 with altered DNAhm, delta beta = 0.06, *padj* = 0.0003; mRNA log2FC = −1.21, *padj* < 1e^−4^) and *SGPP2* (cg27350348 with altered DNAm and DNAhm, DNAm delta beta = −0.06, DNAm *padj* = 0.02, DNAhm delta beta = 0.07, DNAhm *padj* = 0.05; mRNA log2FC = 0.76, *padj* < 1e^−4^). The relations between DNAm, DNAhm, and expression varied according to the gene and the CpG site in question (Supplementary Material, Table S10). For both WT and A30P aSyn LUHMES cells, the overlap between differentially methylated, hydroxymethylated, and expressed genes was not greater than expected by chance (WT *padj* = 0.431, A30P *padj* = 0.171, 10 000 permutations).

**Figure 5:**
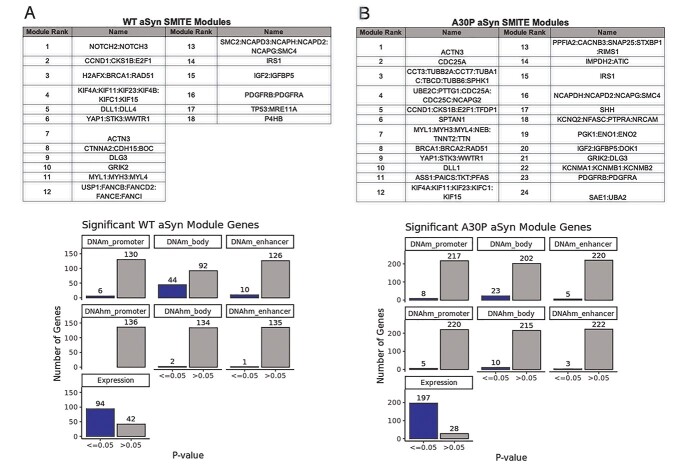
Modules from SMITE analysis were driven by gene expression, with contributions from DNAm and/or DNAhm. (A) Top: Names and ranks of 18 SMITE modules in control vs. WT aSyn LUHMES cells. Bottom: Distribution of significant (*p* ≤ 0.05) and non-significant (*p* > 0.05) SMITE combined *p*-values (Fisher’s method) for each score category, across 136 genes with at least one significant category in control vs. WT aSyn analysis. (B) Top: Names and ranks of 24 SMITE modules in control vs. A30P aSyn LUHMES cells. Bottom: Distribution of significant (*p* ≤ 0.05) and non-significant (*p* > 0.05) SMITE combined *p*-values (Fisher’s method) for each score category, across 225 genes with at least one significant category in control vs. A30P aSyn analysis. DNA(h)m data: *n* = 7 control LUHMES cells and *n* = 8 WT aSyn and A30P aSyn LUHMES cells, expression data: *n* = 3/group (9), H3K4me1 ChIP-seq data: *n* = 3/group. All samples are distinct biological replicates.

We additionally employed SMITE, which captures modules of functionally related genes with changes to at least one of DNAm, DNAhm, or gene expression in each comparison (61). We included H3K4me1 ChIP-seq data from the same LUHMES cells used for DNA(h)m and mRNA analyses in our SMITE workflow, allowing us to consider DNAm and DNAhm changes at enhancers in addition to the default promoter and gene body regions, and weighted the importance of each modification (expression: 0.4; DNAm: 0.35; DNAhm: 0.25). Eighteen modules were identified in WT aSyn cells, encompassing functions including DNA damage repair, cell cycle control, neuronal differentiation, chaperone activity, and cell signaling (insulin, glutamate, and PPAR) (Fig. 5A, Supplementary Material, Fig. S7). Twenty-four modules were identified in A30P aSyn cells, which had similar functions to the WT modules as well as some additional modules related to the urea cycle, sumoylation, PDGF signaling, and K^+^ channel transport (Fig. 5B, Supplementary Material, Fig. S8). Modules from both comparisons were primarily driven by gene expression, with some contributions from DNAm and/or DNAhm (Fig. 5). This effect was consistent even when DNAm or DNAhm was weighted higher in the SMITE model (Supplementary Material, Tables S11,S12).

**Figure 6:**
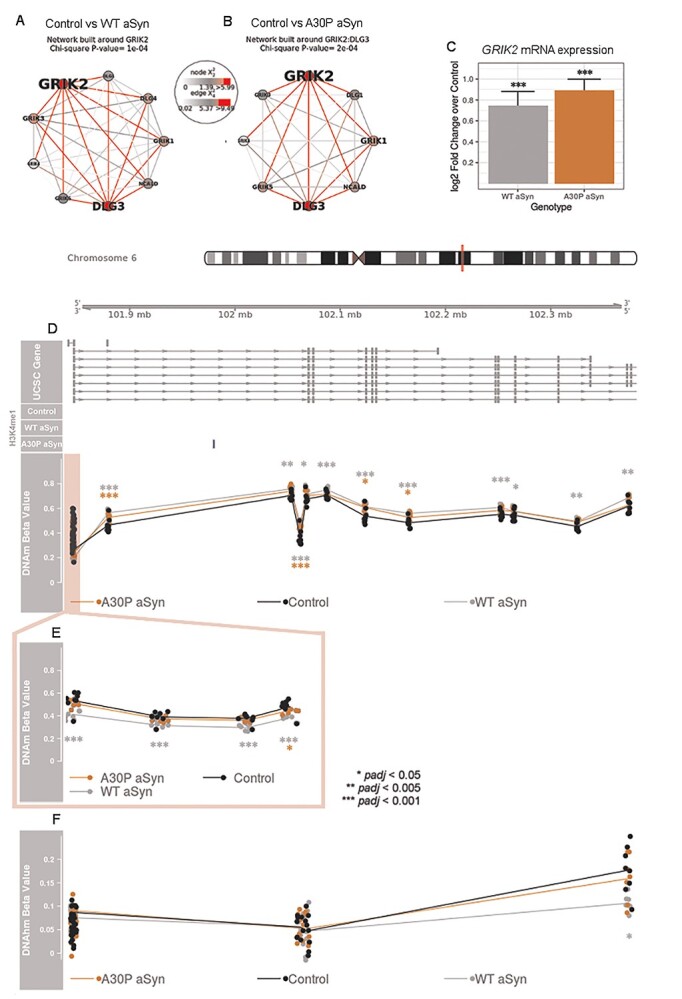
*GRIK2* was differentially methylated, hydroxymethylated, and expressed in LUHMES cells overexpressing either WT or A30P aSyn. (A) GRIK2 protein–protein interaction network showing differential DNAm, DNAhm, and expression in control vs. WT aSyn cells for the underlying genes. (B) GRIK2:DLG3 protein–protein interaction network showing differential DNAm, DNAhm, and expression in control vs. A30P cells for the underlying genes. (C) Relative expression of *GRIK2* mRNA in each genotype, normalized relative to control cells (9). WT aSyn *padj* = 1.46e^−6^, A30P aSyn *padj* = 2.71e^−15^ (*DESeq2* Wald test with Benjamini–Hochberg adjustment). Error bars: SE. (D) Top: UCSC hg19 coordinates are shown, with *GRIK2* transcripts below. Middle: LUHMES cell H3K4me1 consensus peaks are shown in blue. Bottom: DNAm beta values are shown for each sample, colored by genotype. (E) DNAm at the *GRIK2* TSS200 region. (F) DNAhm levels for EPIC array probes across the *GRIK2* gene. *p*-values: *limma* empirical Bayes moderated *t* test with Benjamini–Hochberg adjustment. DNA(h)m data: *n* = 7 control LUHMES cells and *n* = 8 WT aSyn and A30P aSyn LUHMES cells, expression data: *n* = 3/group (9), H3K4me1 ChIP-seq data: *n* = 3/group. All samples are distinct biological replicates.

Glutamate receptor signaling modules discovered using SMITE had more concurrent changes to DNAm, DNAhm, and transcription in both genotypes. Two modules centered around *GRIK2* were each significantly altered in WT and A30P cells, consistent with the direct comparison of differentially (hydroxy)methylated versus differentially expressed genes. *GRIK2* promoter DNAm and mRNA levels were negatively correlated in both genotypes (Fig. 6). Several differentially methylated *GRIK2* promoter CpGs in both genotypes also belonged to a CMR (chr6: 101846707–101 846 916), which contained five individually significant CpGs from the control vs. WT aSyn comparison and one individually significant CpG from the control vs. A30P aSyn comparison.

## Discussion

It is important to elucidate the mechanisms underlying the development and progression of PD in different individuals to develop preventative strategies and treatments. Here, we explored whether DNAm and DNAhm contributed to interindividual differences in PD etiology among carriers of aSyn variants. We assessed the impacts of overexpressing WT or A30P aSyn on genome-wide DNAm and DNAhm patterns in dopaminergic neurons, the primary cell type affected in PD. Our results showed that overexpressing either aSyn variant induced thousands of DNAm changes and hundreds of DNAhm changes in pathways related to PD and neurodegeneration, and that WT aSyn particularly impacted glutamate receptor signaling genes at both epigenetic and transcriptional levels. The distinct characteristics of each aSyn protein may explain why both similar and unique effects on DNA(h)m were observed. This study enhances our understanding of the wide-ranging genomic impacts of different aSyn forms and provides further insights into the possible molecular mechanisms of PD.

The association of aSyn expression with differential DNAm of genes associated with locomotor behavior observed here was consistent with previous reports that aSyn plays a role in dopamine biosynthesis and that dopaminergic neuron activity has impacts on locomotor phenotypes (62,63). The altered DNAm, DNAhm, and expression of neuronal differentiation genes in both genotypes also agreed with previous work in *SNCA* triplication PD patient-derived induced pluripotent stem cells (iPSCs), which indicated reduced expression of genes involved in neuronal differentiation and impaired differentiation capacity associated with aSyn overexpression (64). Several genes identified in our SMITE network analysis have altered epigenetic regulation in the brains of PD patients, including *DLG4* (differentially methylated in PD cingulate gyrus) and *MAGI2* (differentially methylated in the frontal cortex and blood of PD patients, and shows increased H3K27ac in the prefrontal cortex of PD patients) (13,15,17). Glutamate signaling pathway genes have also been implicated in PD, primarily through excessive glutamatergic transmission associated with excitotoxicity in dopaminergic neurons (65). Consistent with these observations, one of the differentially methylated genes shared between WT aSyn-expressing LUHMES neurons and mouse hippocampus was a metabotropic glutamate receptor, and differentially methylated and hydroxymethylated genes in WT aSyn-expressing mice had similar functions to genes with differential methylation, hydroxymethylation, and/or expression in our SMITE analysis. These observations suggested that although DNAm and DNAhm patterns are highly cell type-specific and can change with age and environment, some of the major signaling, transcriptional, and metabolic pathways altered by aSyn expression in this model are more likely to have functional relevance across different physiological contexts, possibly including the human brain. In addition, many of the CpGs identified in site-specific differential (hydroxy)methylation analyses belonged to CMRs, including *SNCA* and *GRIK2*, suggesting that these CpGs are likely to impact regulation of glutamate receptor genes and other pathways affected by aSyn as a unit (58).

Ionotropic and metabotropic glutamate receptor expression are at least in part epigenetically controlled, as transcription of both receptor types is modulated by H3K4 methylation across the human lifespan (66). Signaling cascades activated as a result of glutamate receptor stimulation can also result in gene expression changes, possibly including expression of glutamate receptor genes themselves (67). Therefore, it is possible that aSyn-induced changes in DNAm of the promoters of glutamate receptor genes may be one mechanism underlying the regulation of their expression and subsequent glutamate release, thus facilitating excitotoxicity. It is also possible that existing high levels of glutamate release in the context of aSyn expression resulted in activation of signaling cascades in this study, which altered glutamate receptor expression, and DNAm changes occurred after this expression change. Although it is not possible to determine which of these scenarios is true based on our data, this study provided reasonable support for a role of aSyn-mediated epigenetic deregulation of glutamate receptor expression, with potential downstream impacts on synaptic activity and excitotoxicity. Indeed, preclinical studies have highlighted glutamate receptors as therapeutic targets for PD, and regulation of glutamate signaling genes may be influenced by lifestyle factors, such as dietary exposure to neurotoxins or tea polyphenols (26,68–70). Consistent with our findings, aSyn overexpression was also recently shown to induce glutamate release from mouse neurons and astrocytes *in vitro* and *in vivo* and to activate glutamate receptors (71). While we did not directly examine glutamatergic transmission in this study, our results provide evidence from an epigenetic perspective to expand existing research on the role of glutamate signaling in the etiology and prevention of PD, and support a potential role for aSyn in this pathway.

In addition to these core similarities in the pathways affected by each aSyn variant, WT and A30P aSyn also had unique effects on the DNA methylome and hydroxymethylome. Broadly, many more DNAm and DNAhm changes were seen in WT aSyn cells than in A30P cells in comparison with controls. This was somewhat surprising in light of our previous work, which showed greater effects at the transcriptional level in A30P cells (9). These differences were unlikely to be related to aSyn protein levels, as both WT and A30P LUHMES neurons expressed similar levels of aSyn (9). It was also unexpected that there were almost no changes to non-CpG methylation with aSyn overexpression (one differentially methylated CH probe in WT vs. A30P aSyn cells) (Supplementary Material, Table S3) despite known enrichment of non-CpG methylation in neurons (72,73).

Several mechanisms may explain the greater number of DNAm changes observed in WT aSyn cells than in A30P aSyn cells. First, DNA damage occurs to a greater extent in WT aSyn LUHMES neurons than in A30P aSyn LUHMES neurons, and it is possible that some DNAm changes in WT aSyn cells are a reflection of this damage and/or subsequent repair attempts (9,74). Following DNA damage, protective mechanisms in areas of active transcription maintain DNA repair and restore epigenetic modifications, which may also explain why we observed more DNAm changes in intergenic regions but fewer in intragenic regions than expected by chance in both genotypes (74,75). Second, differences in characteristics of each protein could also explain the greater number of DNAm alterations in WT cells. WT aSyn could influence DNAm through various mechanisms, including binding to membrane-bound G protein-coupled receptors and initiation of signaling cascades; facilitation of SNARE complex assembly at presynaptic membranes, which could allow neurotransmitters to activate signaling cascades at postsynaptic neurons; and binding to DNA (6,8,76). The A30P mutation prevents aSyn from binding to membranes, thus removing some of these avenues of influence (7). Finally, WT aSyn can sequester Dnmt1 from the nucleus in mice, resulting in global loss of DNAm in the brain (77). Differences in DNMT1 sequestration between aSyn variants could reduce the capacity for maintenance of DNAm in a nonspecific manner. aSyn aggregation, which is more likely with the WT protein, has also been shown to increase this sequestration (77). Although A30P aSyn is more likely to be localized to the nucleus than WT aSyn, the ability of A30P aSyn to sequester Dnmt1 has not been assessed (76–78). In addition to influencing DNAm loss in WT aSyn cells and gain in A30P aSyn cells, this potential difference in nuclear aSyn may also affect the amount of DNAm at transcription-associated regions due to its ability to bind DNA (6,76–79).

In contrast to DNAm, the patterns of differential hydroxymethylation were similar between aSyn genotypes, and fewer DNAhm changes were seen at our statistical thresholds. This was expected due to the fetal origin of the LUHMES cell line (43,80). Some of these DNAhm patterns in differentiated LUHMES cells may still be informative for later life stages as certain genes, particularly cell type-specific genes, retain similar brain DNAhm signatures through early development into adulthood (72). The overall increase in DNAhm with aSyn overexpression observed here was consistent with previous reports showing that DNAhm levels are increased in neurons and cerebellar white matter from PD patients (48,49). The opposite direction of change for DNAm and DNAhm at the same loci was also expected, as DNAm must be oxidized for the formation of DNAhm (60). From the results of this study, it is not possible to determine whether this DNAhm was a transient intermediate or represented a stable epigenetic mark; however, correlating DNAhm patterns with gene expression levels has been shown to provide some insight regarding loci where it is associated with transcription (43).

It is important to understand the interplay between DNAm, DNAhm, and gene expression to gain insight into the molecular etiology of PD, and cell culture models, such as LUHMES cells, provide appropriate platforms for concurrent multi-omics profiling. The relatively weak correlations between DNA(h)m changes and expression changes in this study (< 0.1% of genes in either genotype) were expected and were consistent with the literature, where correlations with expression at only 0.6%–15% of CpG sites in blood and up to 0.3% of CpG sites in brain have been reported (81–85). Therefore, it was also unsurprising that there was no significant enrichment for the overlap between differentially methylated, differentially hydroxymethylated, and differentially expressed genes. However, the few loci where transcriptional changes were correlated with DNA(h)m changes may still have either functional relevance for PD or represent potential biomarkers of neurodegeneration-relevant pathways. For the subset of genes with changes to both DNAm and mRNA expression, DNAm changes may have occurred first, thus impacting expression of disease-relevant genes, or the reverse may be true, i.e. DNAm changes may reflect alterations in gene expression (81).

When changes in DNAm precede changes in transcription, DNAm may represent a target for modulation of gene expression to prevent, for example, upregulation of glutamate signaling genes in individuals with aSyn multiplications. Indeed, an epigenetic editing approach using site-directed DNAm via a dCas9-DNMT3A fusion construct was reported previously to successfully reduce aSyn levels in *SNCA*-triplication patient-derived iPSC dopaminergic neurons, demonstrating therapeutic possibilities for manipulation of DNAm in modulating aberrant transcription (86). Conversely, when DNAm changes follow transcriptional changes, DNAm is more likely to represent a biomarker of expression.

Finally, DNAm alterations that did not affect gene expression at this cross-sectional time point may have an impact on expression at later times or upon exposure to particular stimuli (87). Further studies involving exposure of aSyn-expressing neurons to substances and stimuli relevant to PD risk and/or neuroprotection may be useful to examine whether the role of DNA(h)m in the toxicity of aSyn is related to an influence on the ability of the cell to respond appropriately to either risk or protective factors for PD.

The assessment of whether changes in DNA(h)m associated with aSyn overexpression are correlated with transcriptional changes will provide insights regarding whether the roles of cytosine modifications in the toxicity of aSyn are mediated by effects on gene expression. However, it should be noted that this study was not designed to elucidate the mechanisms underlying differential methylation in certain loci in our aSyn-expressing LUHMES neurons. In the general context of the complex and multi-layered regulation of chromatin biology, and given the specific limitations of existing epigenome-wide association study approaches, it is only possible to speculate about the pathways and signals targeting specific CpG loci across the human genome, including the observed alterations in DNAm levels at the endogenous *SNCA* gene itself (33).

This study provided novel insights into the epigenomic impact of aSyn overexpression and set the stage for future studies in samples derived from PD patients. Nevertheless, it should be taken into consideration that integration of the *SNCA* transgene occurred at random positions in the genome in our cells, which could affect DNAm levels surrounding the integration positions. To minimize this variability and represent a range of integration positions, we used eight replicates of each LUHMES cell genotype and seven replicates of control LUHMES cells for EPIC array profiling, with each replicate consisting of a pool of thousands of cells. Cell culture has also been shown to influence DNAm (88–90). We reduced the effects of such variability by using replicates obtained from a range of passages and culture dishes, and regressed out the effects of passage before analysis of differential DNA(h)m. In addition, as expected, determination of DNAhm using a combination of oxidative bisulfite- and bisulfite-converted samples introduced technical noise, which prompted us to reduce the number of sites for DNAhm analysis (91). Finally, the LUHMES cells used in this study are an approximation of dopaminergic neurons in the brains of PD patients, but are subject to the inherent limitations of any *in vitro* cellular model. However, LUHMES cells and other tissue culture models of aSyn overexpression (primarily iPSCs and neuroblastoma lines) have been used successfully in previous studies to uncover biochemical mechanisms of PD, including mitochondrial dysfunction, increased H3 acetylation, and cell–cell transfer of aSyn protein (9,92,93). LUHMES cells provide a useful platform in which to study the biology of aSyn and possibly gain insight into preclinical stages of PD, prior to aSyn aggregation and neurotoxicity on a large scale. As aSyn overexpression has been shown previously to affect cellular physiology even at the neural precursor stage, such studies are still valuable (94). In addition, our LUHMES cells have an advantage over iPSC studies in that the cells are of neural origin, reducing the potential for epigenetic changes associated with iPSC reprogramming (89,90).

Overall, this study demonstrated that WT and A30P aSyn overexpression each had significant impacts on the DNA methylome of human dopaminergic neurons, and that only a small proportion of these DNAm changes occur concomitantly with DNAhm and changes in expression of neurodegeneration-related genes. This study contributes to our understanding of the molecular genetic etiology of PD and lays groundwork for future studies to investigate whether epigenetic and transcriptional changes associated with increased expression or mutations in aSyn may be reversible by modification of environmental and/or lifestyle factors.

## Materials and Methods

### Cell culture

LUHMES cells were a kind gift from Prof. Marcel Leist and were cultured and differentiated as described previously (9,80,95); the methods below are reproduced from (9) with permission. Briefly, proliferating cells were cultured in Advanced Dulbecco’s modified Eagle’s medium/F12 (DMEM/F12, Gibco, Thermo Fisher Scientific, Waltham, MA, USA) supplemented with N2 (Gibco), 2 mM l-glutamine (Gibco), and 40 ng/mL recombinant basic fibroblast growth factor (bFGF, R&D Systems Inc., Minneapolis, MN, USA), in pre-coated flasks (Corning Inc., Corning, NY, USA) with 50 μg/mL PLO (Sigma-Aldrich, St. Louis, MO, USA), at 37°C in a humidified 5% CO_2_ atmosphere. Differentiation was achieved by replacing the proliferating medium with DMEM/F12 supplemented with N2 (Gibco), 2 mM l-glutamine (Gibco), 1 μg/mL tetracycline (Sigma-Aldrich), 1 mM dibutyryl cAMP (cAMP; Sigma-Aldrich), and 2 ng/mL recombinant human GDNF (R&D Systems). After 2 days in culture (pre-differentiation), cells were trypsinized and seeded into plates pre-coated with 50 μg/mL PLO and 1 μg/mL fibronectin (Sigma-Aldrich). On day 5 of differentiation, one third of the culture medium was replaced with fresh medium. Cells were harvested after 8 days of differentiation.

### Generation of aSyn-expressing LUHMES cells

Expression of aSyn in LUHMES cells was achieved as described previously (9,95); the methods below are reproduced from (9) with permission. Full-length human aSyn c-DNA (SNCA, NM_000345) and A30P aSyn were subcloned into the second-generation bicistronic lentiviral vector, pWPI (Tronolab, Lausanne, Switzerland), under the control of the chicken β-actin (CBA) promoter. All cloned sequences were verified by direct sequencing (StarSeq, Mainz, Germany). The pWPI lentiviral vector containing only the internal ribosomal entry site (IRES)-green fluorescent protein (GFP) cassette was used as a positive control for infection in all experiments. Second-generation lentiviral particles were generated as described previously (96). Briefly, 293 T cells were transiently co-transfected with the modified transfer vector plasmids and second-generation packing system (Tronolab). Supernatant was collected 48-h post-transfection, concentrated by PEG-it Virus Precipitation Solution (System Biosciences, Palo Alto, CA, USA), and resuspended in Panserin 402 (Pan-Biotech, Aidenbach, Germany). Lentiviral vector titers were measured quantitatively by qPCR using primer sequences specific for the woodchuck hepatitis virus post-transcriptional regulatory element (WPRE), as described previously (97). To generate cells expressing aSyn, proliferating LUHMES cells were infected with equimolar amounts of IRES-GFP and WT aSyn-IRES-GFP or A30P aSyn-IRES-GFP lentivirus. Cells positive for green fluorescence were selected by FACS (FACSCalibur; BD Biosciences, Franklin Lakes, NJ, USA).

### Immunocytochemistry

Immunocytochemistry was performed as described previously (9,95); the methods below are reproduced from (9) with permission. Cells were grown on coverslips and fixed at different time points of differentiation with 4% paraformaldehyde for 40 min at room temperature (RT). After washing with 1× phosphate buffered saline (PBS), the cells were permeabilized with 0.5% Triton/PBS for 15 min, and finally blocked with 1.5% normal goat serum (NGS)/PBS (blocking buffer) for 1 h at RT. Cells were incubated with anti-aSyn antibody (mouse, 1:1000; BD Transduction Laboratories, Franklin Lakes, NJ, USA) and anti-TH antibody (rabbit, 1:1000; Millipore, Billerica, MA, USA) for 3 h. The cells were then washed with 1× PBS and probed for 2 h at RT with secondary antibodies (rabbit or mouse, 1:1000 Alexa 488 and 1:1000 Alexa 555; Life Technologies, Carlsbad, CA, USA) diluted in blocking buffer. Finally, the nuclei were stained with DAPI and mounted with Mowiol (Sigma-Aldrich). The images were acquired on a Leica 6000B microscope (Leica Microsystems, Wetzlar, Germany).

### DNA extraction and bisulfite conversion

Cell pellets were thawed on dry ice and homogenized with a 20G needle in Qiagen Buffer RLT Plus (Qiagen, Valencia, CA, USA) with β-mercaptoethanol. DNA extractions were performed with a Qiagen AllPrep DNA/RNA Mini Kit in accordance with the manufacturer’s instructions. DNA quantity and purity were assessed by spectrophotometry.

DNA samples of 1.5 μg were split into two equal aliquots of 750 ng for sodium bisulfite (BS) conversion or oxidative bisulfite (oxBS) conversion using the NuGEN TrueMethyl oxBS Module (NuGEN Technologies, San Carlos, CA, USA). One aliquot per sample was treated with the oxidation protocol, while the second aliquot was subjected to mock oxidation. All samples were then sodium bisulfite converted according to the manufacturer’s instructions.

### DNA methylation and DNA hydroxymethylation analysis

Aliquots of 750 ng per sample of BS- or oxBS-converted DNA were run on Infinium HumanMethylationEPIC (EPIC) BeadChips (Illumina, San Diego, CA, USA) according to the manufacturer’s instructions, producing data for 853 307 CpG and 2880 CNG sites (where N represents any other nucleotide) (data available at https://www.ncbi.nlm.nih.gov/geo/ with accession No. GSE181126). Beta values were generated from raw intensity signals using GenomeStudio software (Illumina) and exported into R ver. 3.6 (R Foundation for Statistical Computing, Vienna, Austria) for data analysis. Filtering was performed to remove 59 internal SNP control probes and 43 254 cross-hybridizing probes (98). The pfilter function in the wateRmelon package was additionally used to remove sites with a detection *p* > 0.05 in 1% of samples (7685) and sites with a bead count < 3 in 5% of samples (1626) (98). This left 813 589 EPIC probes in the final dataset. The dasen function in wateRmelon was used to normalize the oxBS and BS data separately (99). Batch effects from chip, position, and passage were removed using the ComBat function in the SVA package (100). The hmC values were calculated by subtracting the normalized oxBS beta values from the normalized BS beta values. A threshold for hmC detectability (3.6%) was calculated using the 95% quantile of negative hmC values generated after subtraction; all probes with a mean hmC level below this threshold were discarded (91).

Differential methylation between control (*n* = 7), WT aSyn (*n* = 8), and A30P aSyn (*n* = 8) cells was calculated by linear regression using limma with the model DNA(h)m ~ Genotype (101). Individual sites were considered significant at FDR ≤ 0.05 and |delta beta| ≥ 0.05 (calculated by subtracting the mean beta value across all replicates of the group in question from the reference group, e.g. WT aSyn beta − Control beta). The skewness.norm.test function from the normtest R package was used to assess delta beta skewness, with 1000 Monte Carlo simulations (https://cran.r-project.org/web/packages/normtest/normtest.pdf).

CMRs were defined separately in pre-processed DNAm and DNAhm data from LUHMES neurons using the cmr function from the CoMeBack R package with a maximum distance of 1 kb, minimum Pearson’s correlation coefficient of 0.4, and minimum of two CpGs per CMR (58). Composite betas were calculated with the cmr_comp function, using the default method (weighted average of the first principal component of CMR probe DNA(h)m levels). As in site-specific analysis, linear modeling with limma was applied to the DNAm and DNAhm composite betas, with one composite beta value and one CpG probe ID representing each CMR. The overlap in differentially (hydroxy)methylated probes from the CMR and site-specific analyses was permuted 10 000 times by randomly sampling a set of CpG probes from the preprocessed EPIC DNAm or DNAhm data the same size as the number of significant site-specific probes, randomly sampling a set of representative CMR probes from the DNAm or DNAhm CMRs the same size as the number of significant CMRs, and calculating how many iterations had greater or smaller overlap than the real value.

All code is publicly available at github.com/samschaf/LUHMES.

### Pyrosequencing

1 μL of BS or oxBS-converted DNA per 30-μL reaction was used to perform PCR with HotStarTaq DNA Polymerase (Qiagen) for 45 cycles with an annealing temperature of 58°C. PCR samples were visualized on a 1% agarose gel post-amplification to ensure integrity.

Pyrosequencing assays were designed with PyroMark® Assay Design 2.0 software (Qiagen) (Supplementary Material, Table S5). 10 μL of PCR reaction mixture, 70 μL of binding buffer, and 12 μL of sequencing reaction mix per sample were prepared with PyroMark® Gold Q96 Reagents (Qiagen) according to the manufacturer’s instructions. Sequencing was performed with the PyroMark® Q96 ID (Qiagen). Differential DNAm/DNAhm between groups was assessed by Welch’s two-sample *t* test (two-tailed), using the t.test() function in R.

### Generation of aSyn-expressing mice and genome-wide DNAm/DNAhm profiling

Generation of WT aSyn-expressing mice and DNA extraction were performed as described previously (14). Hippocampal DNA from four WT and four transgenic mice raised under standard housing conditions to the age of 12 months was profiled by RRBS for genome-wide DNAm and DNAhm. RRBS libraries were prepared following the protocol of Gu *et al.* (102), using 400 ng of genomic DNA per sample. 0.4 ng of unmethylated phage lambda DNA per sample was added during the *Msp*I digestion step as a control for bisulfite conversion efficiency, and *Msp*I reactions were performed with incubation at 37°C for 16 h. Digested DNA was end-repaired, and then purified using AMPure XP magnetic beads (Beckman Coulter Life Sciences, Fullerton, CA, USA) at 2× concentration, and 0.1 μM methylated adapters (IDT, Coralville, IA, USA) were used in ligation reactions. Ligated DNA was purified with 1.5× magnetic beads and quantified using a Qubit dsDNA HS Assay Kit (Thermo Fisher Scientific). Eight samples per lane were pooled in equimolar fashion, and each pool was split into two aliquots for oxBS and BS conversion using the NuGEN TrueMethyl oxBS Module (NuGEN Technologies). One aliquot was subjected to oxidation, while the second aliquot was subjected to mock oxidation; the bisulfite conversion protocol was then applied to both. Libraries were then amplified by PCR with Pfu Turbo Cx HotStart DNA polymerase for 14 cycles (Agilent Technologies, Santa Clara, CA, USA). Size selection (200–550 bp) was performed using a 0.55× AMPure beads wash (Agencourt Bioscience, Beverly, MA, USA) followed by two 1.2× washes for cleanup. The final libraries were eluted in 25 μL of Qiagen EB Buffer (Qiagen), and quality was assessed by PCR, spectrophotometry, fluorometry, and DNA high-sensitivity chip (Agilent Technologies). 75 bp paired-end sequencing was conducted with Illumina HiSeq 2500 by the Michael Smith Genome Sciences Centre (Vancouver, bc, Canada).

BSMAP was used to align reads to the mm10 and phage lambda genomes and to calculate methylation ratios (103). Sequence data was assessed for quality before and after alignment using FastQC software (https://www.bioinformatics.babraham.ac.uk/projects/fastqc/). Methylation ratio data was then read into R ver. 3.6.3 and filtered for: a minimum read depth of 10×; a maximum read depth of the 99.9% quantile of coverage; and sites covered in all samples. oxBS beta values were used to represent DNAm, while BS − oxBS values were used to represent DNAhm, with the 95% quantile of negative hmC values generated after subtraction used as a detectability threshold (91). We removed cytosines with ≤ 10% variability between groups to reduce the multiple testing burden for differential DNA(h)m analysis.

Differential DNA(h)m analysis was conducted with the betaRegression function from the BiSeq R package, using beta-binomial regression with the Wald test for significance (104). The *p*-values were adjusted for multiple comparisons using the Benjamini–Hochberg method. Cytosines were considered differentially methylated at *padj* ≤ 0.05 and |delta beta| ≥ 0.1.

To determine whether more genes differentially methylated in WT aSyn-expressing LUHMES neurons were also differentially methylated in mice than expected by chance, we first subset the RRBS data to genes differentially methylated in LUHMES cells. We considered the ‘hit list’ of differentially methylated cytosines in mice as the number of cytosines within this subset passing *padj* ≤ 0.05 and |delta beta| ≥ 0.1. Next, we randomly selected a set of cytosines the same size as this ‘hit list’ from the subsetted RRBS background, and determined the genes to which they mapped. The number of differentially methylated genes in the random selection was compared to the number of differentially methylated genes in the ‘hit list.’ This process was repeated 10 000 times in each of the DNAm and DNAhm datasets. Enrichment and depletion *p*-values were calculated by dividing the number of iterations with more or fewer differentially methylated genes than the real number by the number of permutations.

### Gene ontology enrichment

Genes were assigned to CpGs if they belonged to the longest UCSC RefGene transcript annotated to each site, resulting in 30 325 unique coding and non-coding gene annotations for the DNAm dataset and 23 190 unique gene annotations for the DNAhm dataset. Gene Ontology enrichment analysis was performed with the over-representation (ORA) method in ermineR 1.0.1.9, using differentially methylated genes identified with the effect size and significance thresholds described above as input (59). Genes that were differentially methylated or hydroxymethylated in both WT aSyn and A30P aSyn cells in either direction were removed from the input list and analyzed separately. All code is publicly available at github.com/samschaf/LUHMES.

### RNA sequencing and differential expression analysis

RNA-seq of LUHMES cells used for multi-omic integration analysis in this study was described previously (9,95) (data available at https://www.ncbi.nlm.nih.gov/geo/ with accession No. GSE89115); the methods below are reproduced from (9) with permission. Total RNA from differentiated LUHMES cells was extracted and purified using an RNeasy mini kit (Qiagen) in accordance with the manufacturer’s instructions. Three biological replicates were used for RNA-seq experiments. Sequencing and RNA quality analyses were performed as described previously (105). Briefly, RNA quality was assessed using RNA 6000 Nano chips run on a 2100 Bioanalyzer (Agilent Technologies). Libraries were prepared using a TruSeq RNA Sample Preparation v2 kit (Illumina). The library quality was checked using High-Sensitivity DNA chips on a Bioanalyzer (Agilent Technologies). The sample concentration was measured with a Qubit dsDNA HS Assay Kit and adjusted to 2 nM before sequencing (50 bp) on a HiSeq 2000 sequencing system (Illumina) in accordance with the manufacturer’s instructions.

Differential gene expression analysis was performed as described previously (105). Briefly, RNA-seq data were aligned to the genome using STAR with the default options, which generated mapping files (BAM format). Differential expression read counts were generated using featureCounts, and samples were compared for differential expression using DESeq2 (106,107). Genes with *p* ≤ 0.05 and mean read count ≥10 were considered to be differentially expressed.

Transcript-level analysis of *SNCA* mRNA was performed with Salmon v1.6.0 and the hsapiens gentrome (genome + transcriptome) derived from Gencode v39 using the following parameters: —numGibbsSamples 20 —seqBias —gcBias —validateMappings (108). Scaling abundances to final values was done in R with tximeta v1.10.0 (109). Differential expression of transcripts was calculated with the fishpond R package (110).

### Chromatin immunoprecipitation sequencing (ChIP-seq)

Chromatin and DNA were cross-linked by adding formaldehyde to the culture dish to a final concentration of 1% and incubating for 10 min at 25°C. The cross-linking reaction was quenched with glycine at a final concentration of 0.125 M at 25°C. Cells were then collected, washed with cold PBS, and pelleted by centrifugation at 900 rcf for 5 min at 4°C. Cell pellets were resuspended in RIPA SDS buffer (140 mM NaCl, 1 mM EDTA at pH 8.0, 1% Triton X-100, 0.1% sodium deoxycholate, 10 mM Tris-Cl at pH 8.0, 1% SDS) supplemented with Roche complete protease inhibitors, incubated at 4°C for 10 min, and then used for shearing in Covaris® instrument. The sheared chromatin was cleared by centrifugation at 16000 rcf for 5 min and an aliquot was used to check DNA size on an Agilent 2100 Bioanalyzer (Agilent Technologies). Immunoprecipitation was performed when DNA was sheared to fragments of 150–300 bp.

For chromatin immunoprecipitation (ChIP), samples were diluted 10× in IP buffer (150 mM NaCl, 1% NP-40, 0.5% sodium deoxycholate, 50 mM Tris–HCl, pH 8.0, 2 mM EDTA) supplemented with Roche complete protease inhibitors (Roche, Basel, Switzerland), pre-cleared with protein A magnetic beads (Dynabeads; Invitrogen, Carlsbad, CA, USA) for 1 h at 4°C, and then used for immunoprecipitation with histone modification-specific ChIP-grade antibody (anti H3 monomethyl K4 antibody, ab8895; Abcam, Cambridge, UK). After overnight incubation at 4°C, protein A magnetic beads were added and incubated for 2 h at 4°C. Beads were then washed twice with cold IP buffer with 0.1% SDS and protease inhibitors, 3× with wash buffer (100 mM Tris–HCl, pH 8.0, 500 mM LiCl, 1% v/v NP-40, 1% w/v sodium deoxycholate, 2 mM EDTA), and 2× with TE. After the last wash, supernatant was removed and beads were resuspended in 1 mM Tris (pH 8.0) and RNase A (0.1 μg/μL), and incubated 30 min at 37°C. To reverse the cross-linking, beads were incubated overnight at 65°C in 100 mM Tris–HCl (pH 8.0), 20 mM EDTA, 2% SDS, and proteinase K (0.5 μg/μL). The DNA was purified with SureClean (Bioline, Taunton, MA, USA) and DNA concentration was measured with a Qubit dsDNA HS Assay Kit (Thermo Fisher Scientific).

Libraries were generated with 3 ng of ChIPed DNA using a NEBNext Ultra II DNA Library Prep Kit for Illumina (New England Biolabs, Ipswich, MA, USA), size was determined with Agilent Bioanalyzer DNA high-sensitivity (Agilent Technologies), and sequencing was performed on an Illumina NovaSeq 6000 instrument (Illumina) according to the manufacturer’s instructions. Read quality of ChIP-seq data was assessed using FastQC (v0.11.5) and reads were aligned to the human genome version 38 using Bowtie2 (v2.0.2) (https://www.bioinformatics.babraham.ac.uk/projects/fastqc/, 111). MACS2 (v2.1.2) was used to call broad peaks from BAM files (112).

### Multi-omic integration

The overlap between differentially methylated, hydroxymethylated, and expressed genes in each comparison was permuted 10 000 times to test whether it was greater or smaller than expected by chance. For this purpose, a set of EPIC probes from the preprocessed DNAm data the same size as the number of differentially methylated probes was randomly selected, a set of EPIC probes from the preprocessed DNAhm data the same size as the number of differentially hydroxymethylated probes was randomly selected, and a set of genes from the RNA-seq data the same size as the number of differentially expressed genes was randomly selected. The gene-level overlap between randomly sampled DNAm, DNAhm, and RNA-seq data was then calculated, and this number was compared to the real number to calculate enrichment and depletion *p*-values.

DNAm, DNAhm, and RNA-seq data were also integrated using SMITE in R ver. 3.6, with adjusted *p*-values as significance input, delta betas as effect size input for DNAm and DNAhm, and log2FC values as effect size input for RNA-seq (62). Genomic regions were split into enhancers (± 5000 bp from H3K4me1 ChIP-seq peaks), promoters (± 1000 bp from TSS), and gene bodies (remaining regions). DNAm and DNAhm *p*-values were weighted across genomic regions according to their significance using Stouffer’s method, creating a combined *p*-value for each region. The *p*-values from all modifications were then used to create weighted gene-level scores, with application of the following weights: expression, 0.4; enhancer DNAm, 0.125; promoter DNAm, 0.125; body DNAm, 0.1; enhancer DNAhm, 0.125; promoter DNAhm, 0.125; body DNAhm, 0.1. Weights were chosen to return modules likely to contain differentially expressed genes, with equal probability of contribution from differential DNAm and/or differential DNAhm (total weight of 0.25 for each methylation type). Enhancers and promoters were weighted slightly higher than gene bodies due to the higher likelihood that DNAm at these regions would influence gene expression. Alternative SMITE models with equal weights for each modification, DNAm weighted highest, DNAhm weighted highest, or DNAm and expression weighted higher than DNAhm were also tested, and produced overall similar results (Supplementary Material, Tables S11,S12). Scores were annotated to a REACTOME protein–protein interaction network, and a spin-glass algorithm was used to identify modules that had altered DNAm, DNAhm, and expression for each comparison. All code is publicly available at github.com/samschaf/LUHMES.

## Supplementary Material

Supplementary_Material_revision3_ddac104
