# Supplementary material for: Alpha-synuclein overexpression induces epigenomic dysregulation of glutamate signaling and locomotor pathways
Source: Hum Mol Genet. 2022 May 14;31(21):3694–714. doi: 10.1093/hmg/ddac104 (PMC9616577; doi:10.1093/hmg/ddac104)
Supplement: Supplementary_Material_revision3_ddac104 [file supplementary_material_revision3_ddac104.pdf]

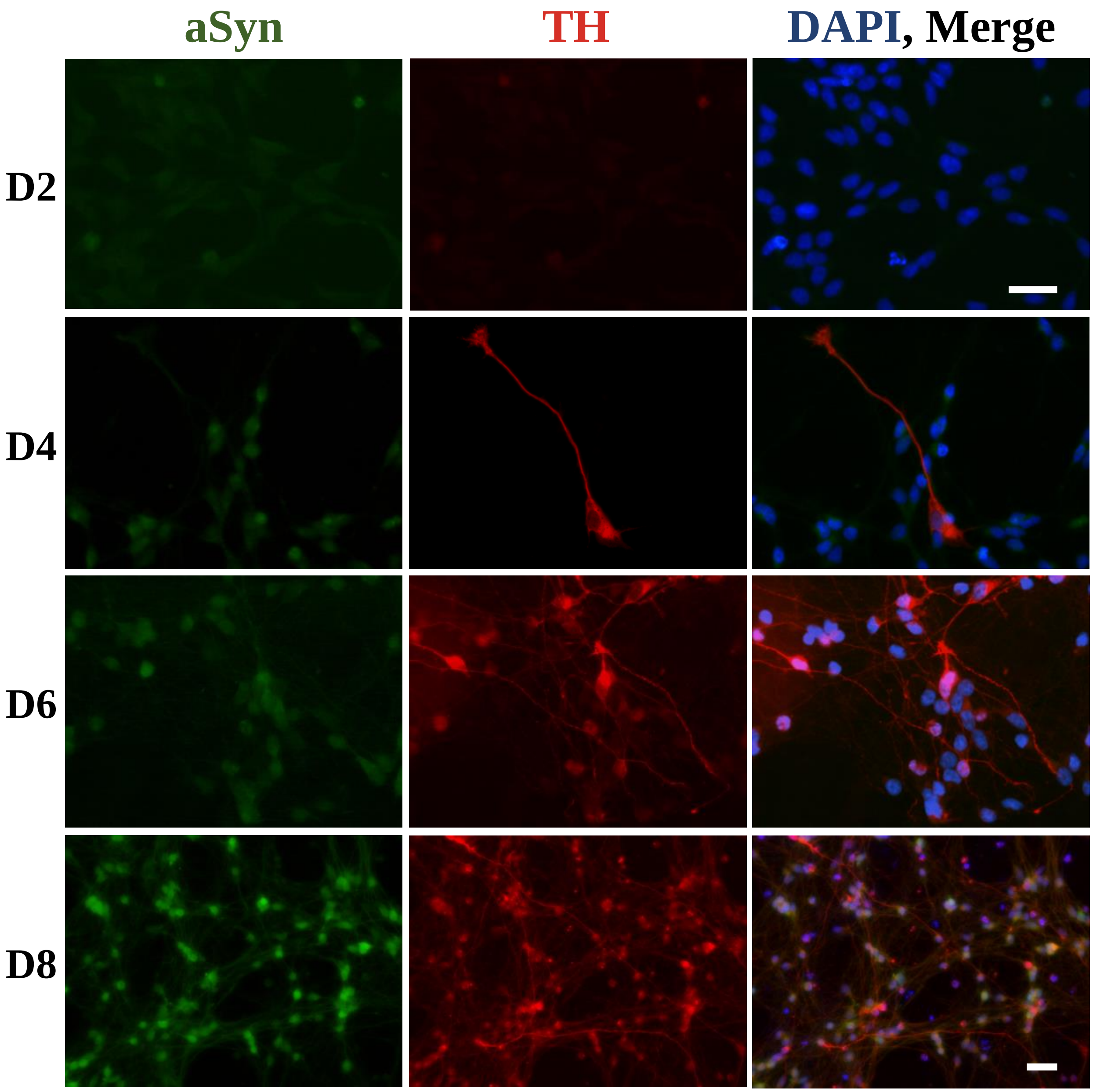

Supplementary Figure 1. Increases in levels of aSyn and TH in LUHMES cells during differentiation. LUHMES cells were differentiated on pre-coated glass coverslips for up to 8 days. At different time points, the cells were fixed and stained for aSyn and TH. Scale bar: 30 µm.

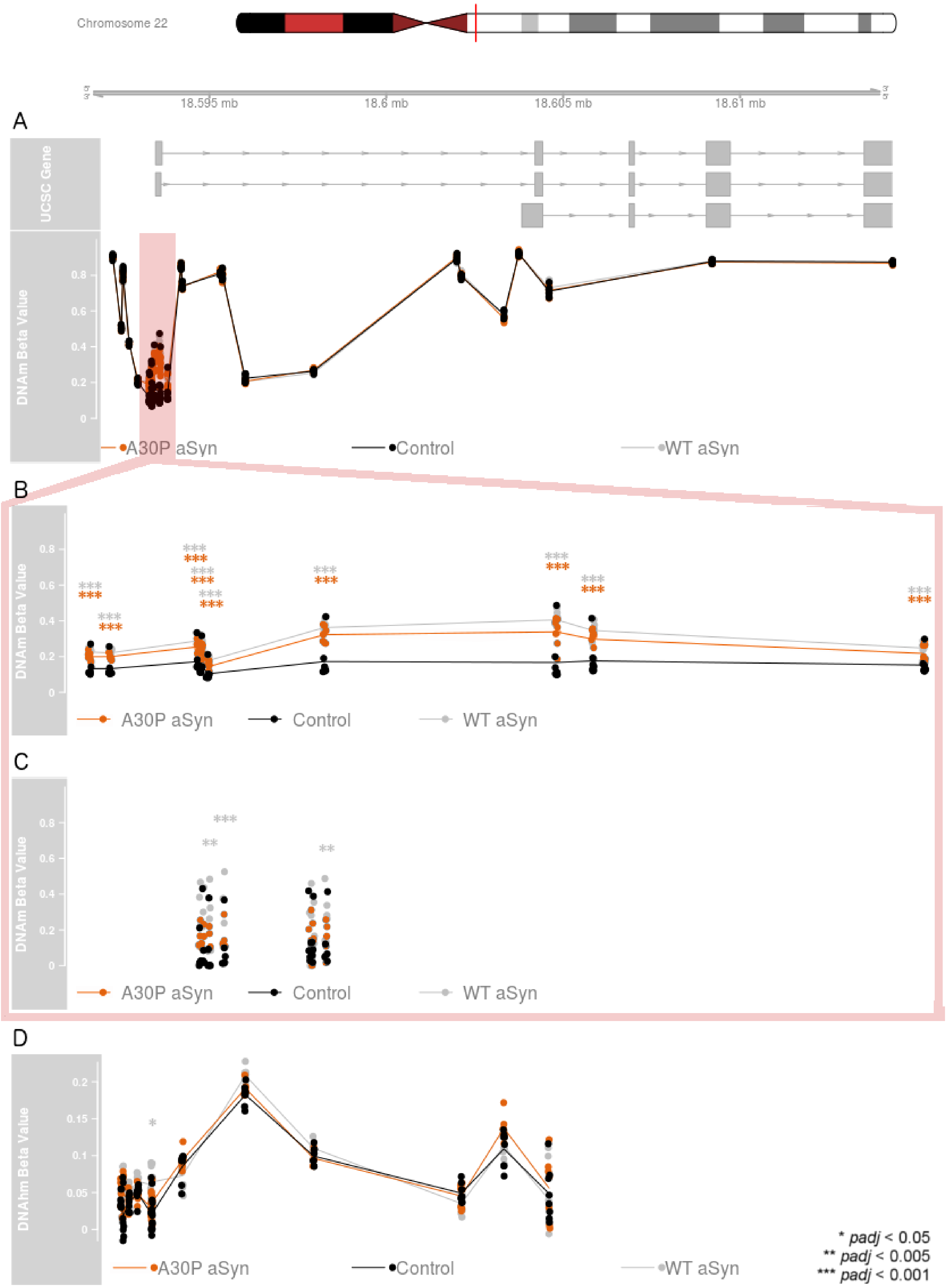

Chromosome 22
18.595 mb
18.6 mb
18.605 mb
18.61 mb
A
UCSC Gene
DNAm Beta Value
A30P aSyn
Control
WT aSyn
B
DNAm Beta Value
A30P aSyn
Control
WT aSyn
C
DNAm Beta Value
A30P aSyn
Control
WT aSyn
D
DNAhm Beta Value
A30P aSyn
Control
WT aSyn
* padj < 0.05
** padj < 0.005
*** padj < 0.001

Supplementary Figure 2. ***TUBA8*** **was differentially methylated in both LUHMES genotypes and differentially hydroxymethylated in WT aSyn cells.** Top: UCSC hg19 coordinates are shown, with TUBA8 transcripts below; gray boxes represent exons, lines represent introns. Bottom: Beta values are shown for each sample, colored by genotype. Black: control cells (n = 7 biological replicates); gray: WT aSyn cells (n = 8); orange: A30P aSyn cells (n = 8). (A) DNAm levels for all EPIC array probes across the *TUBA8* gene. (B) Differentially methylated region of *TUBA8*. *** *padj* < 0.001 (*limma* empirical Bayes moderated t test with Benjamini–Hochberg adjustment). (C) DNAm levels at seven CpG sites in the *TUBA8* differentially methylated region measured by oxidative bisulfite pyrosequencing. Control: n = 6 biological replicates, WT aSyn: n = 8, A30P aSyn: n = 8. Left to right, p-values: 0.0075 ($t = -5.53$, df = 12), 0.0022 ($t = -6.47$, df = 4.33), 0.0075 ($t = -5.36$, df = 3.68; Welch's two-sample t test, two- tailed). (D) DNAhm levels for all EPIC array probes across the *TUBA8* gene. * *padj* = 0.049 (*limma* empirical Bayes moderated t test with Benjamini–Hochberg adjustment).

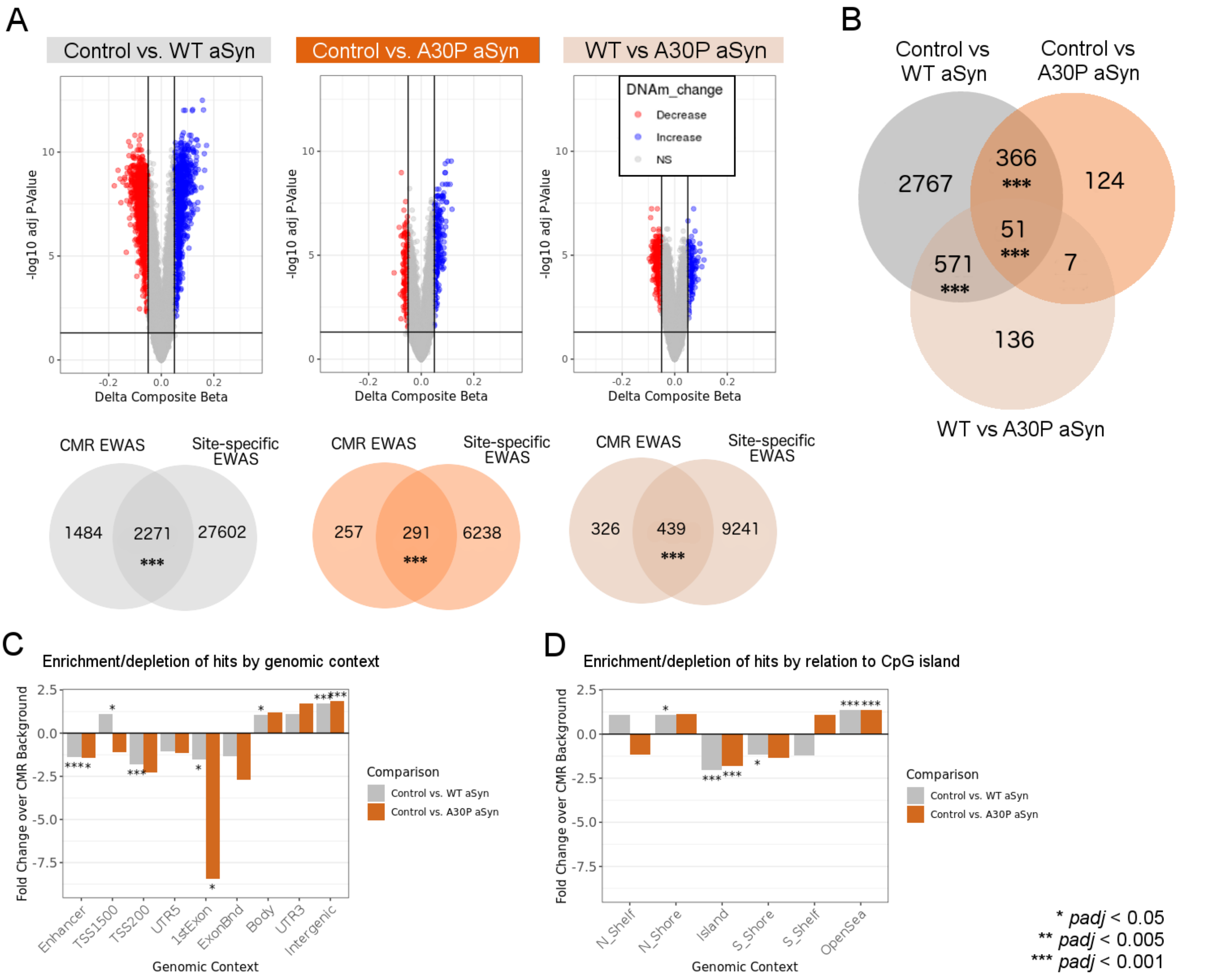

Supplementary Figure 3. Overexpression of WT and A30P mutant aSyn altered regional DNA methylation patterns in dopaminergic neurons. (A) Top: Volcano plots comparing co-methylated region (CMR) DNAm patterns between control (*n* = 7 biological replicates) and WT aSyn (*n* = 8) LUHMES, control and A30P aSyn (*n* = 8) LUHMES, and WT aSyn and A30P aSyn LUHMES. Colored points passed thresholds of delta composite beta ≥ 0.05 and *padj* ≤ 0.05 (*limma* empirical Bayes moderated *t* test with Benjamini–Hochberg adjustment). Bottom: Number of representative CMR CpG probes and individual CpG sites significant from CMR and site-specific epigenome-wide association study (EWAS) approaches (overlap *padj* < 0.001, 10,000 permutations). (B) Number of differentially methylated CMR probes unique to each comparison and probes shared between comparisons (*padj* < 0.001, 10,000 permutations). (C) Relative enrichment/depletion of differentially methylated CMR probes across genomic contexts, permuted against CMR background (10,000 iterations). (D) Relative enrichment/depletion of differentially methylated CMR probes by relation to CpG islands, permuted against CMR background (10,000 iterations).

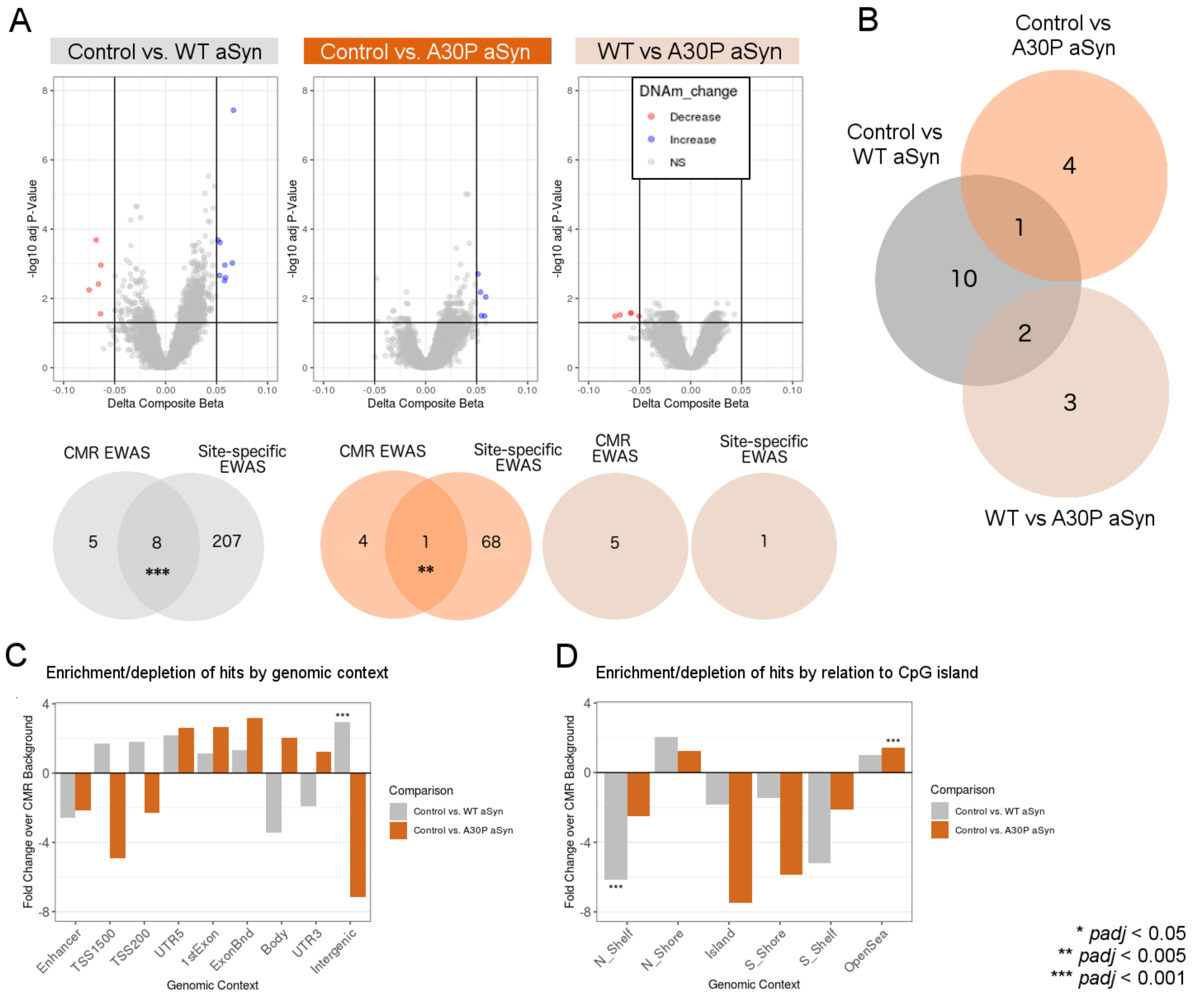

Supplementary Figure 4. Overexpression of WT and A30P mutant aSyn altered regional DNA hydroxymethylation patterns in dopaminergic neurons. (A) Top: Volcano plots comparing co-methylated region (CMR) DNAhm patterns between control (n = 7 biological replicates) and WT aSyn (n = 8) LUHMES, control and A30P aSyn (n = 8) LUHMES, and WT aSyn and A30P aSyn LUHMES. Colored points passed thresholds of delta composite beta ≤ 0.05 and *padj* ≤ 0.05 (*limma* empirical Bayes moderated t test with Benjamini–Hochberg adjustment). Bottom: Number of representative CMR CpG probes and individual CpG sites significant from CMR and site-specific epigenome-wide association study (EWAS) approaches (*** overlap *padj* < 0.001, ** overlap *padj* < 0.005, 10,000 permutations). (B) Number of differentially hydroxymethylated CMR probes unique to each comparison and probes shared between comparisons. (C) Relative enrichment/depletion of differentially hydroxymethylated CMR probes across genomic contexts, permuted against CMR background (10,000 iterations). (D) Relative enrichment/depletion of differentially hydroxymethylated CMR probes by relation to CpG islands, permuted against CMR background (10,000 iterations).

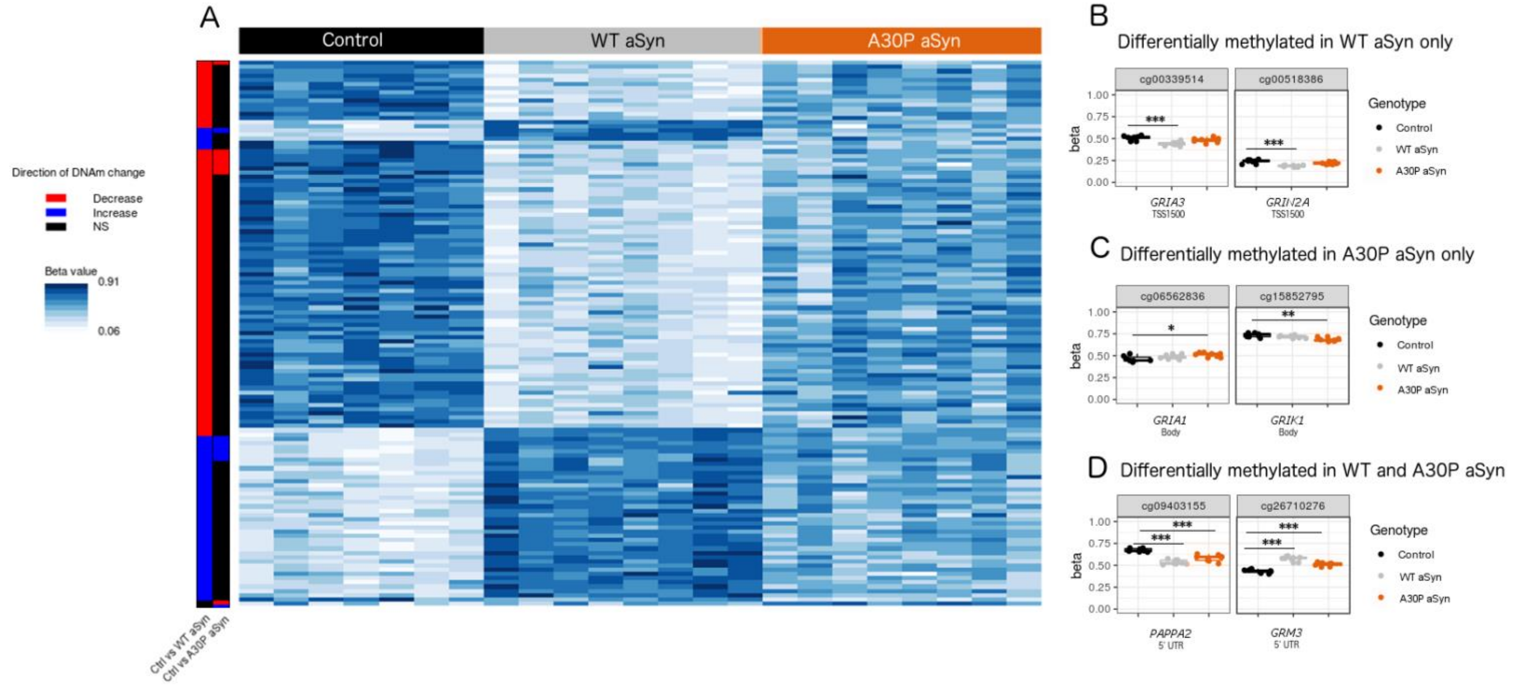

Supplementary Figure 5. Differentially methylated sites associated with glutamate receptor signaling genes in WT and A30P aSyn cells. (A) Heat map showing beta values for all probes annotated to GO:0007215 (glutamate receptor signaling pathway) that were differentially methylated in either control (n = 7 biological replicates) vs. WT (n = 8) or control vs. A30P (n = 8) aSyn analyses. Row labels, left to right: Probes that passed significance thresholds (absolute delta beta ≥ 0.05 and *padj* ≤ 0.05, *limma* empirical Bayes moderated *t* test with Benjamini–Hochberg adjustment) in control vs. WT aSyn comparison (decreased DNAm in WT aSyn red; increased DNAm in WT aSyn blue; non-significant: black). Probes that passed significance thresholds in control vs. A30P aSyn comparison (decreased DNAm in A30P aSyn: red; increased DNAm in A30P aSyn: blue; non-significant: black). (B) Representative examples of 111 total glutamate signaling-related CpG sites differentially methylated only in WT aSyn cells (GRIA3 *padj* = 2.48e^{-4}, GRIN2A *padj* = 5.79e^{-6}). (C) All glutamate signaling-related CpG sites differentially methylated only in A30P aSyn cells (GRIA1 *padj* = 0.014, GRIK1 *padj* = 0.0013). (D) Representative examples of 16 glutamate signaling-related CpG sites differentially methylated in both genotypes (PAPPA2 WT aSyn *padj* = 2.57e^{-7}, PPAPPA2 A30P aSyn *padj* = 2.50e^{-4}, GRM3 WT aSyn *padj* = 1.06e^{-8}, GRM3 A30P aSyn *padj* = 1.13e^{-4}). Black: control cells; gray: WT aSyn cells; orange: A30P aSyn cells. * *padj* < 0.05, ** *padj* < 0.005, *** *padj* < 0.001 (*limma* empirical Bayes moderated *t* test with Benjamini–Hochberg adjustment for multiple comparisons).

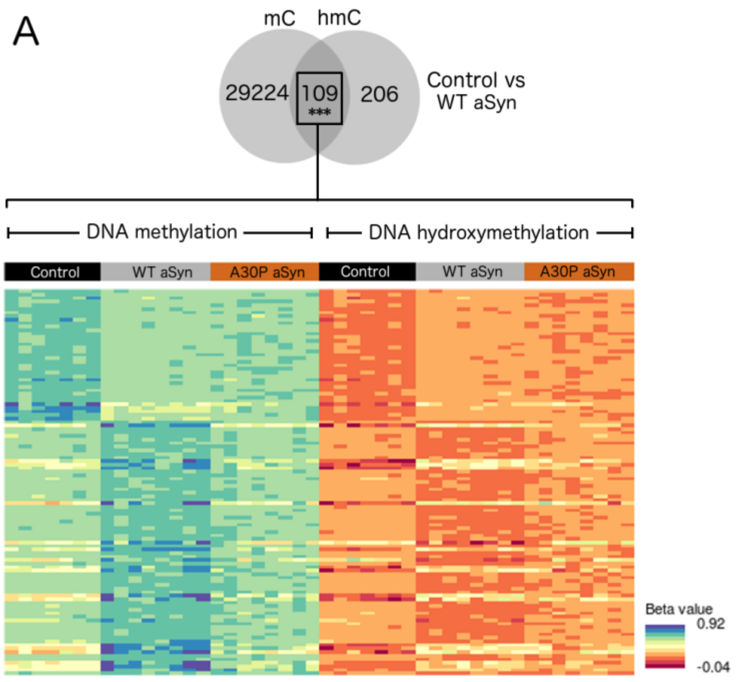

Supplementary Figure 6. DNAm and DNAhm levels of hits in both modifications. Heat maps showing beta values for all probes differentially methylated and hydroxymethylated in each comparison (absolute delta beta ≥ 0.05 and *padj* ≤ 0.05, *limma* empirical Bayes moderated *t* test with Benjamini–Hochberg adjustment for multiple comparisons). Red: low beta value; yellow: medium beta value; blue: high beta value. (A) DNAm and DNAhm levels of 109 hits with changes in both modifications in control ($n = 7$ biological replicates) vs. WT aSyn ($n = 8$). (B) DNAm and DNAhm levels of 34 hits with changes in both modifications in control vs. A30P aSyn ($n = 8$). *** *padj* < 0.001 (1,000 permutations).

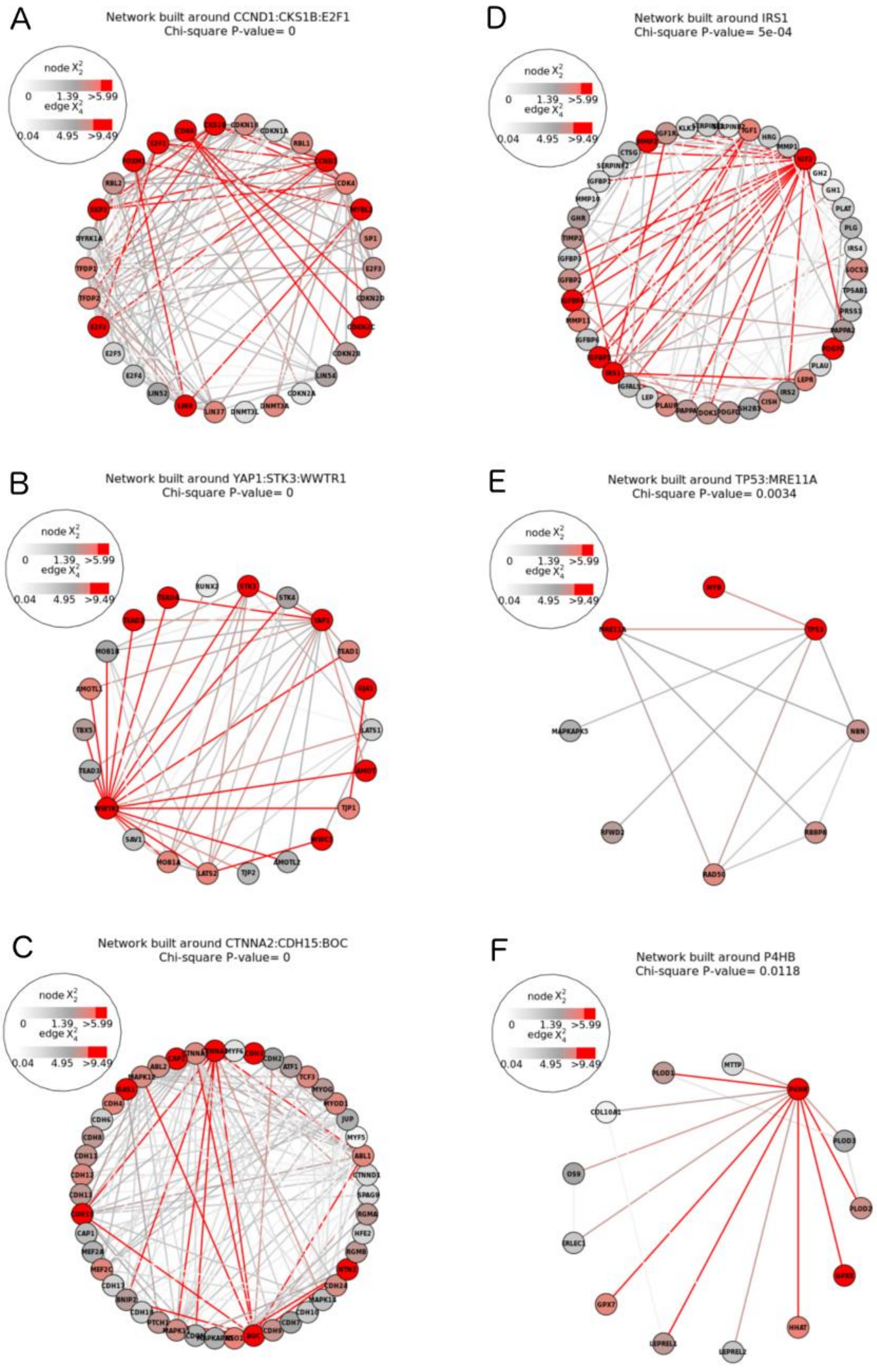

Supplementary Figure 7. SMITE modules altered in WT aSyn cells. Red fill: high scoring gene; red line: high scoring interaction (average score between genes). One representative module of a total of 18 is shown for each of (A) cell cycle regulation, (B) apoptosis, (C) neuron development, (D) insulin signaling, (E) DNA damage repair, and (F) chaperone activity.

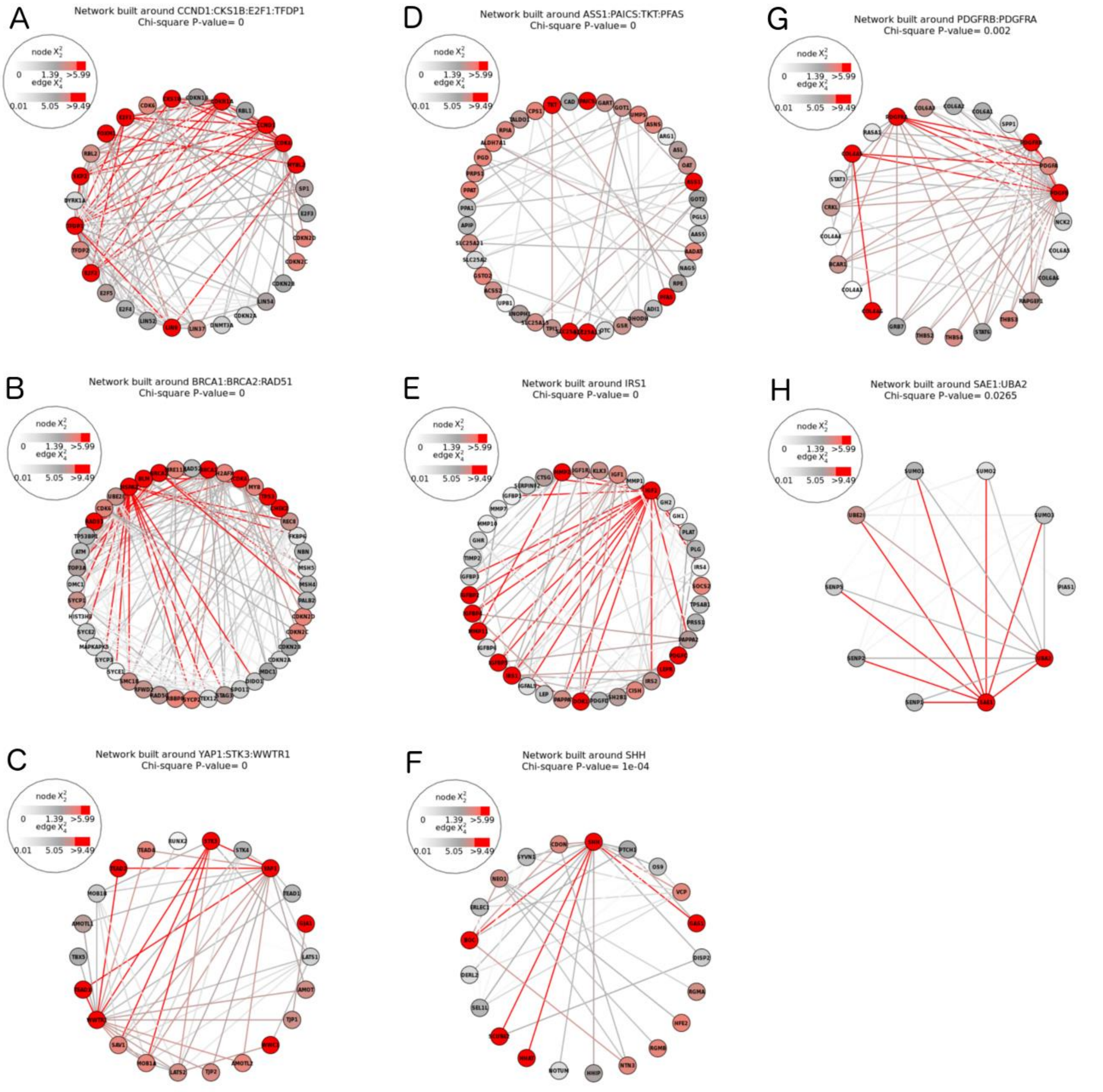

Supplementary Figure 8. SMITE modules altered in A30P aSyn cells. Red fill: high scoring gene; red line: high scoring interaction (average score between genes). One representative module of a total of 24 is shown for each of (A) cell cycle regulation, (B) DNA damage repair, (C) apoptosis, (D) urea cycle, (E) insulin signaling, (F) neuron development, (G) PDGF signaling, and (H) sumoylation.

Supplementary Table 1. Differential expression of *SNCA* mRNA by transcript.

Transcript	Control vs WT aSyn		Control vs A30P aSyn		WT vs A30P aSyn	
	log2FC	Adjusted p-value	log2FC	Adjusted p-value	log2FC	Adjusted p-value
ENST00000336904.7	-0.39288486	0.786719072	-0.283054959	0.855510894	-0.096006448	0.989109836
ENST00000394986.5	0.762370566	0.283303082	0.838966482	0.28820401	-0.0251302	0.976725988
ENST00000394991.8	0.512211849	0.324155462	1.045014969	6.62E-06	-0.501703384	0.95644647
ENST00000502987.5	-0.088731201	0.951987876	0.333731284	0.841189316	-0.347263295	0.95699789
ENST00000505199.5	8.087727806	7.39E-06	7.987599899	6.62E-06	0.115435707	0.969374289
ENST00000506244.5	9.21412088	7.39E-06	9.521769968	6.62E-06	-0.298630021	0.95644647
ENST00000506691.1	6.164828861	7.39E-06	6.204709696	6.62E-06	-0.033699748	0.969374289
ENST00000508895.5	1.097336656	7.39E-06	1.257481167	6.62E-06	-0.173133585	0.963809844
ENST00000674129.1	-0.642761814	0.555244954	0.240498634	0.972865971	-0.752482968	0.95644647

Supplementary Table 5. Pyrosequencing assays.

**Primer name**	**Primer sequence (5’ → 3’)**	**Sequence to analyze (5’ → 3’)**	**Length (bp)**
TUBA8_F1	AGTTGGGGTTAAGAAGGTTGTTTT	n/a	n/a
TUBA8_R1	AACTACCCCCCACATCACAATAC*		
TUBA8_A1_S1	TGGTAATAGGGTAGTTTTTT	TYGYGGGYGTAGTTTTYGTTG	21

Supplementary Table 10. Genes with differential expression, DNA methylation, and DNA hydroxymethylation in WT aSyn vs. control LUHMES. DB: delta beta.

**gene**	**Expression**		**DNA methylation**				**DNA hydroxymethylation**			
	**mRNA log2FC**	**mRNA adjP**	**DNAm probe**	**DNAm feature**	**DNAm DB**	**DNAm adjP**	**DNAhm probe**	**DNAhm feature**	**DNAhm DB**	**DNAhm adjP**
			cg06325209	TSS200	0.07	0.00				
			cg00457087	1stExon;5'UTR	0.06	0.00				
			cg13420177	5'UTR	0.06	0.00				
			cg21140079	5'UTR	0.06	0.00				
DDR2	-1.57	0	cg17496788	TSS1500	0.05	0.00	cg21140079	5'UTR	-0.06	0.05
			cg24753760	TSS1500;5'UTR; 1stExon	-0.17	0.00				
			cg24432193		0.14	0.00				
			cg13080565	TSS1500;5'UTR; 1stExon	-0.13	0.00				
			cg04708601		0.12	0.00				
			cg18063869		0.11	0.00				
			cg22541254	TSS1500;5'UTR; 1stExon	-0.10	0.00				
			cg06247406	TSS1500;5'UTR; 1stExon	-0.10	0.00				
			cg05942459	TSS1500;5'UTR; 1stExon	-0.09	0.00				
GRIK2	0.75	0	cg11976671		0.09	0.00	cg10769416	NA	-0.08	0.04

			cg10769416		0.09	0.00				
			cg12321434		0.07	0.00				
			cg09834689		0.06	0.01				
			cg21673964	5'UTR;TSS1500	0.06	0.02				
			cg00135293		0.06	0.00				
			cg25135233		0.05	0.02				
			cg23666299		0.05	0.01				
WDR76	-0.71	0	cg01494789	TSS200;5'UTR	0.06	0.00	cg01494789	TSS200;5'UTR	-0.07	0.03
			cg13504432	5'UTR	-0.17	0.00				
			cg13787649	3'UTR;5'UTR	-0.16	0.00				
			cg20336210	5'UTR	-0.16	0.00				
			cg22166425	5'UTR	-0.13	0.00				
			cg17192235	5'UTR	-0.12	0.00				
			cg24604417	5'UTR	-0.12	0.00				
			cg07983668	TSS1500	-0.09	0.00				
			cg10042864	5'UTR	-0.08	0.00				
			cg12764348	5'UTR	0.08	0.00				
			cg08620695	5'UTR	-0.06	0.00				
PARP8	-0.53	0	cg24707825	5'UTR	-0.05	0.00	cg12764348	5'UTR	-0.06	0.02
			cg27396284	5'UTR	0.18	0.00				
			cg18590693		0.14	0.00				
			cg14420059		0.13	0.00				
			cg27321369	5'UTR	0.13	0.00				
			cg15802249	TSS1500;5'UTR	0.12	0.00				
			cg14566882	TSS1500;5'UTR	0.11	0.00				
			cg25406767	TSS1500	0.11	0.00				
			cg26996097	TSS1500;5'UTR	0.11	0.00				
			cg14973347	TSS1500	-0.11	0.00				
			cg01464878	5'UTR	0.11	0.00				
			cg22544467	5'UTR	0.10	0.00				
			cg23050544	TSS1500;5'UTR	0.10	0.00				
			cg10121647		0.10	0.00				
			cg00332048	TSS1500	-0.09	0.00				
			cg10676164		0.09	0.00				
			cg24458314		0.09	0.00				
			cg17761265		0.09	0.00				
			cg08488089	TSS1500	0.08	0.00				
			cg19641966	5'UTR	0.08	0.00				
			cg09048590		0.08	0.00				
			cg27452159	5'UTR	0.08	0.00				
			cg03161886		0.07	0.00				
			cg08285151	TSS1500	-0.07	0.00				
HDAC9	0.82	0	cg16245593		0.07	0.00	cg17227395	NA	0.05	0.04

			cg11993578		0.07	0.00				
			cg17739473	5'UTR	0.07	0.00				
			cg23580915		-0.07	0.00				
			cg04049937		0.06	0.00				
			cg08582594		0.06	0.00				
			cg05865493		0.06	0.00				
			cg12210898		0.06	0.00				
			cg17227395		-0.05	0.00				
			cg20738553	5'UTR	0.05	0.02				
			cg04837104		0.11	0.00				
			cg11631644	TSS1500	0.08	0.00				
			cg19683410		0.07	0.00				
TSPEAR	-0.96	0	cg27251694		0.06	0.01	cg12061357	TSS200	0.06	0.00
			cg18584042	TSS200	-0.16	0.00				
			cg22097777	TSS1500	-0.15	0.00				
			cg09506037	TSS1500	-0.14	0.00				
			cg26428069	TSS1500	-0.14	0.00				
			cg25409638		-0.13	0.00				
			cg02191986	TSS1500;5'UTR	-0.13	0.00				
			cg19414227		-0.12	0.00				
			cg18916494		-0.11	0.00				
			cg18471021	5'UTR	-0.11	0.00				
			cg25153505	5'UTR	-0.11	0.00				
			cg22687497		-0.11	0.00				
			cg02506226	TSS1500	-0.11	0.00				
			cg09587456		-0.11	0.00				
			cg08471449	TSS1500	-0.10	0.00				
			cg00827382	TSS1500	-0.10	0.00				
			cg02440797	TSS1500	-0.10	0.00				
			cg20514322	TSS200	-0.08	0.00				
			cg17081914	TSS1500	-0.07	0.00				
			cg14471282	TSS200	-0.07	0.00				
			cg16208848	5'UTR	-0.07	0.00				
			cg19917530		-0.07	0.00				
			cg25930602	TSS1500	-0.07	0.00				
			cg16328548	5'UTR	-0.07	0.00				
			cg19676227		-0.07	0.00				
			cg06389574		0.06	0.01				
			cg06504046		-0.06	0.00				
			cg25500795	5'UTR	-0.06	0.00				
			cg07469205		-0.06	0.01				
			cg15928974	TSS1500;5'UTR	-0.06	0.00				
NCKAP5	-1.40	0	cg23072408		-0.06	0.00	cg15354803	NA	-0.06	0.04

cg20443798	TSS1500;3'UTR	-0.06	0.00	

Supplementary Table 11. Number of significant SMITE module genes per data type in WT aSyn vs. control LUHMES under different weights.

Model	Data type		Weight	Total weight	Number of significant module genes ($p < 0.05$)
Expression-weighted	DNA methylation	Enhancer	0.125	0.35	10
		Promoter	0.125		6
		Body	0.1		44
	DNA hydroxymethylation	Enhancer	0.075	0.25	1
		Promoter	0.075		0
		Body	0.1		2
	Expression		0.4	0.4	94
Equal-weighted	DNA methylation	Enhancer	0.12	0.33	12
		Promoter	0.12		6
		Body	0.09		45
	DNA hydroxymethylation	Enhancer	0.1	0.33	2
		Promoter	0.1		0
		Body	0.13		2
	Expression		0.34	0.34	107
DNAm-weighted	DNA methylation	Enhancer	0.15	0.4	12
		Promoter	0.15		6
		Body	0.1		45
	DNA hydroxymethylation	Enhancer	0.075	0.25	2
		Promoter	0.075		0
		Body	1		2
	Expression		0.35	0.35	102
DNAhm-weighted	DNA methylation	Enhancer	0.1	0.25	11
		Promoter	0.1		6
		Body	0.05		44
	DNA hydroxymethylation	Enhancer	0.12	0.4	2
		Promoter	0.12		0
		Body	0.16		2
	Expression		0.35	0.35	105
DNAm- and expression-weighted	DNA methylation	Enhancer	0.15	0.4	11
		Promoter	0.15		6
		Body	0.1		45
	DNA hydroxymethylation	Enhancer	0.06	0.2	1
		Promoter	0.06		0
		Body	0.08		2
	Expression		0.4	0.4	98

Supplementary Table 12. Number of significant SMITE module genes per data type in A30P aSyn vs. control LUHMES under different weights.

Model	Data type		Weight	Total weight	Number of significant module genes ($p < 0.05$)
Expression-weighted	DNA methylation	Enhancer	0.125	0.35	5
		Promoter	0.125		8
		Body	0.1		23
	DNA hydroxymethylation	Enhancer	0.075	0.25	3
		Promoter	0.075		5
		Body	0.1		10
	Expression		0.4	0.4	197
Equal-weighted	DNA methylation	Enhancer	0.12	0.33	5
		Promoter	0.12		8
		Body	0.09		19
	DNA hydroxymethylation	Enhancer	0.1	0.33	5
		Promoter	0.1		10
		Body	0.13		10
	Expression		0.34	0.34	159
DNAm-weighted	DNA methylation	Enhancer	0.15	0.4	4
		Promoter	0.15		3
		Body	0.1		20
	DNA hydroxymethylation	Enhancer	0.075	0.25	5
		Promoter	0.075		5
		Body	1		11
	Expression		0.35	0.35	185
DNAhm-weighted	DNA methylation	Enhancer	0.1	0.25	6
		Promoter	0.1		8
		Body	0.05		21
	DNA hydroxymethylation	Enhancer	0.12	0.4	5
		Promoter	0.12		5
		Body	0.16		11
	Expression		0.35	0.35	171
DNAm- and expression-weighted	DNA methylation	Enhancer	0.15	0.4	6
		Promoter	0.15		8
		Body	0.1		22
	DNA hydroxymethylation	Enhancer	0.06	0.2	4
		Promoter	0.06		3
		Body	0.08		11
	Expression		0.4	0.4	188
